# Supplementary material for: Stable coexistence and transport of lytic phage infections with migrating bacterial hosts
Source: Sci Adv. 2026 Oct 21;12(43):eaef9265. doi: 10.1126/sciadv.aef9265 (PMC13685945; doi:10.1126/sciadv.aef9265)
Supplement: Supplementary file 1 [file sciadv.aef9265_sm.pdf]

Science Advances
AAAS

Supplementary Materials for

Stable coexistence and transport of lytic phage infections with migrating bacterial hosts

Jacopo Marchi *et al.*

Corresponding author: Jacopo Marchi, jmarchi@umd.edu; Joshua S. Weitz, jsweitz@umd.edu; Remy Chait, r.chait@exeter.ac.uk

Sci. Adv. **12**, eaef9265 (2026)
DOI: 10.1126/sciadv.aef9265

The PDF file includes:

Figs. S1 to S22
Supplementary Text
Table S1
Legends for movies S1 to S10
References

Other Supplementary Material for this manuscript includes the following:

Movies S1 to S10

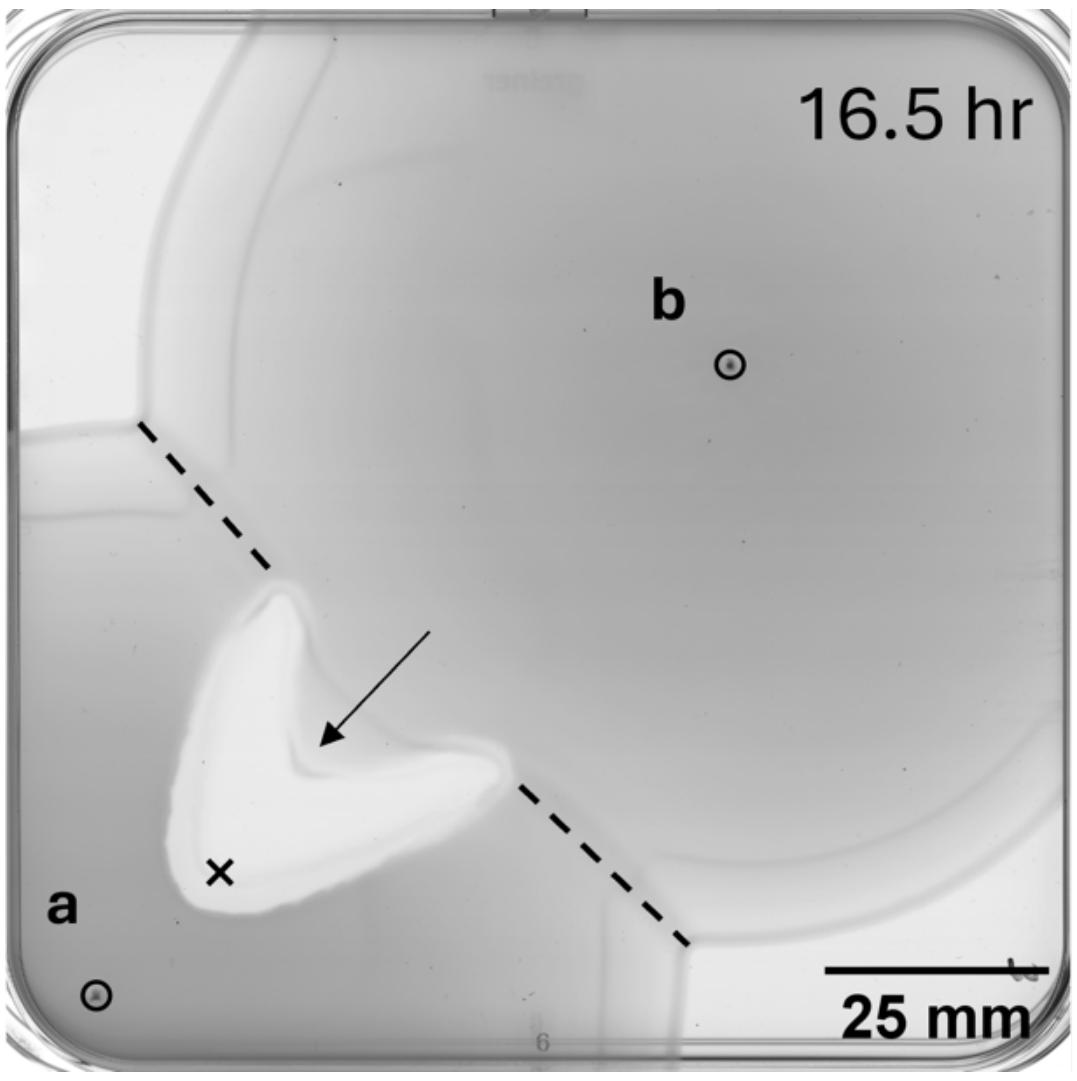

Supplementary Figure 1. A phage-protected but infection-free region can exist within a phage invasion zone. Two *E. coli* W1485(F8) colonies (a,b), migrate outwards through LB swim agar from locations marked by ◦. Colony a, encountering T7 phage at location × develops a clear phage protected zone in the advancing front. As migration halts on collision of colonies a and b (dashed line), the front advancing from b penetrates between the infected wings and into (arrow) the phage invasion zone without lysis before being halted at the periphery, illustrating that phage infection and production can be restricted to the invasion zone edges.

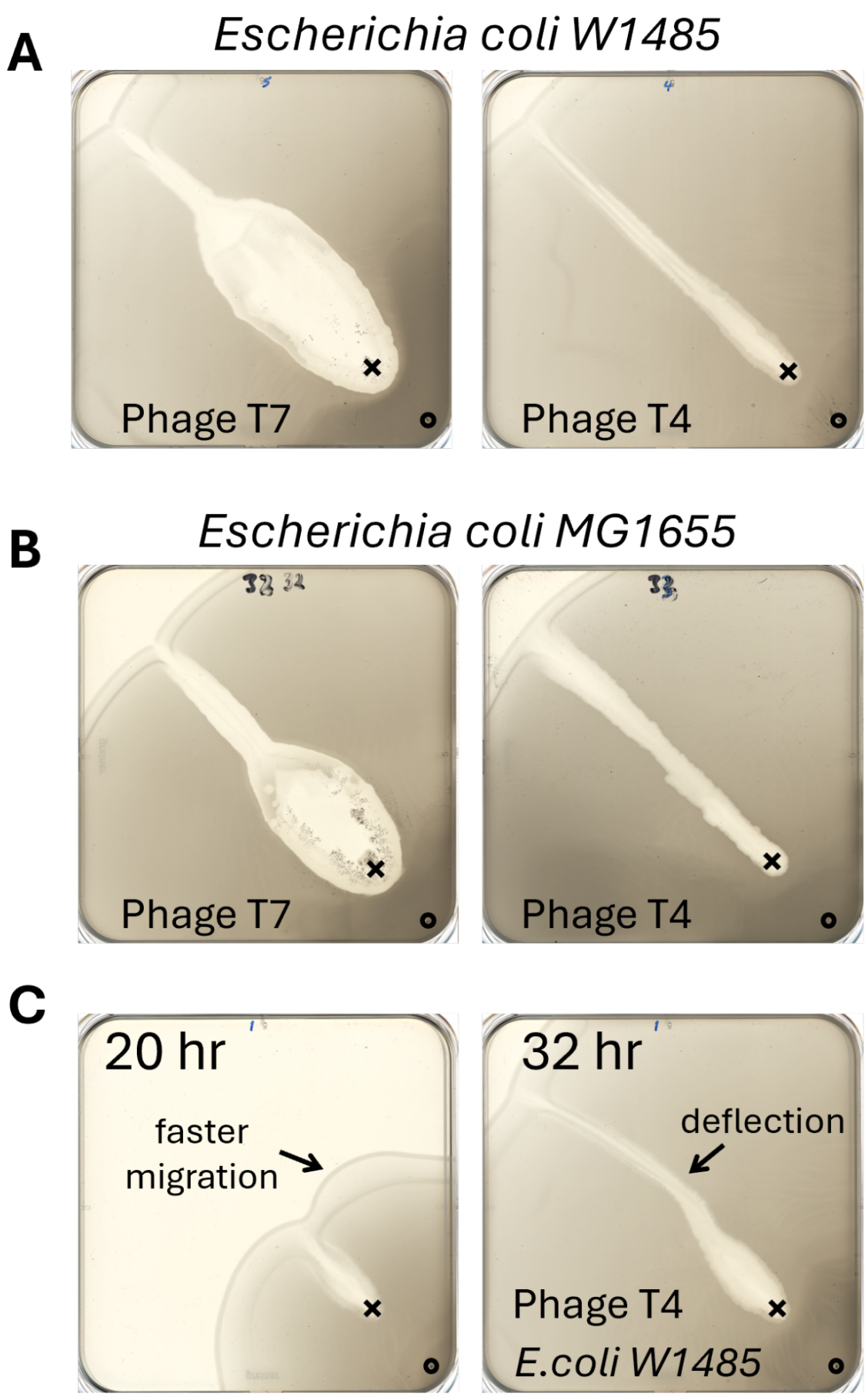

Supplementary Figure 2. Phage T7 and T4 PhASERs on *E. coli* strains W1485 and MG1655**.** *Escherichia coli* strains **(A)** W1485 and **(B)** MG1655 migrating through 12cm square LB agar swim plates both form PhASERs on encountering phages T7 (left) and T4 (right). Host cells and phages are inoculated at locations **o** and **x**, respectively. **(C)** Local emergence of faster migrating cells appears first as a spreading bulge in an *E. coli* W1485 front at 20hr (left), which deflects the T4 PhASER (right). Agar concentrations are 0.25% in **A,C** and 0.3% in **B**. Swim assays are performed at 30C as otherwise described in Methods.

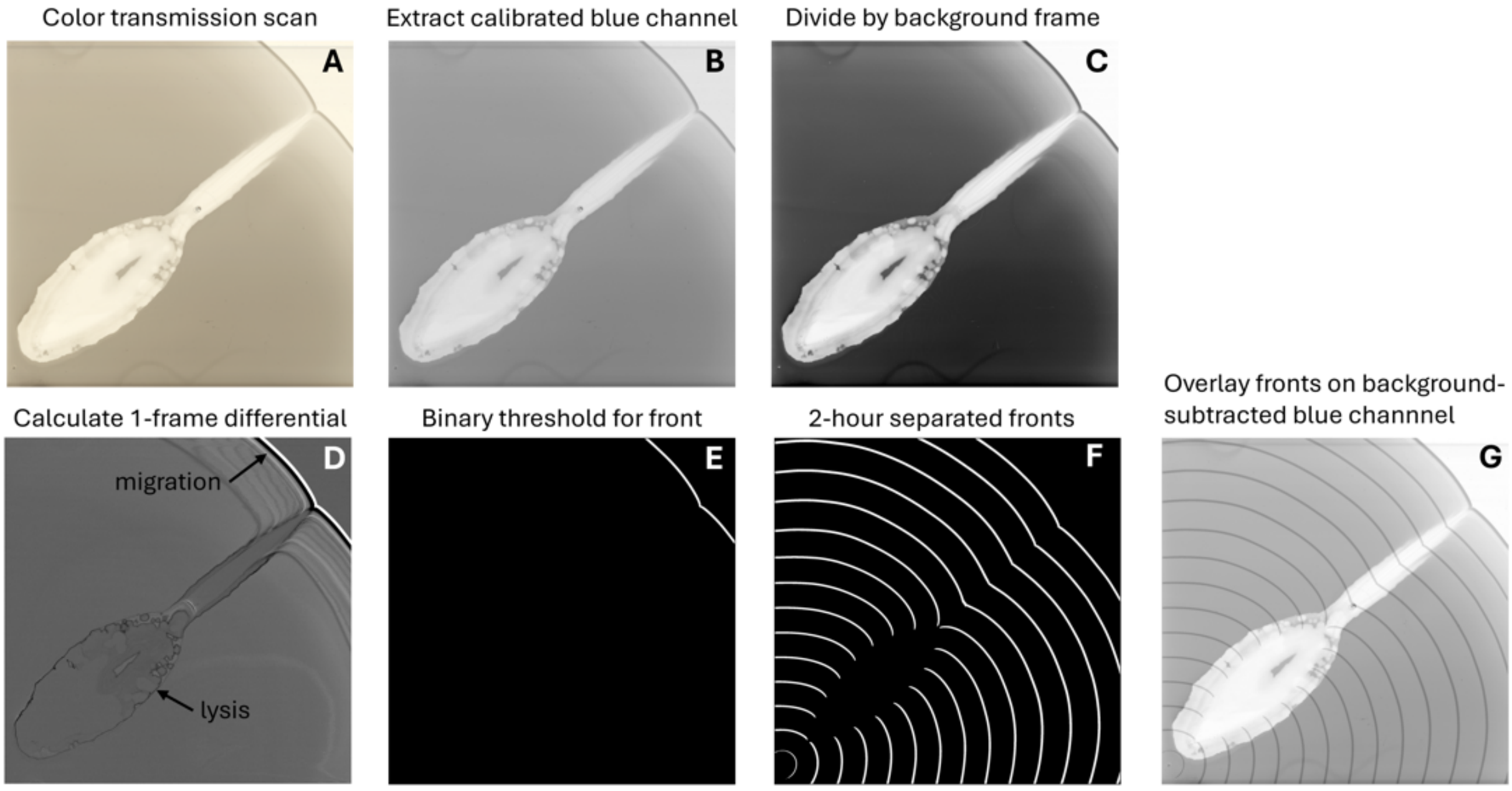

Supplementary Figure 3. Sample image processing steps for timelapse scans of migrating bacteria intersecting phage inocula. Images are processed to highlight spatial cell density and change due to cell growth, migration and lysis. For *E. coli* W1485((F9) interacting with phage T7 (as Figure 3), a scanned transmitted image sequence **(A)** is separated into colour channels. The blue channel **(B)** is calibrated to cell concentration (Supplementary Figure 9). The blue channel is normalized **(C)** by division by the first (background) frame to reduce lighting inhomogeneities. Single frame differential images **(D)** are obtained by subtracting consecutive frames and reveal adjacent light/dark bands of migrating fronts, light background cell growth, and dark edges indicating cell killing. Binary thresholding **(E)** captures the migrating bacterial front. Maximum projection of 2 hour separated fronts **(F)** reveals the position and trajectory of the interaction across time, and can be overlaid on cell image (**G**, Figure 3). Elements of this analysis (**A,C,D,E**) throughout a timelapse sequence are shown in Supplementary Movie 10.

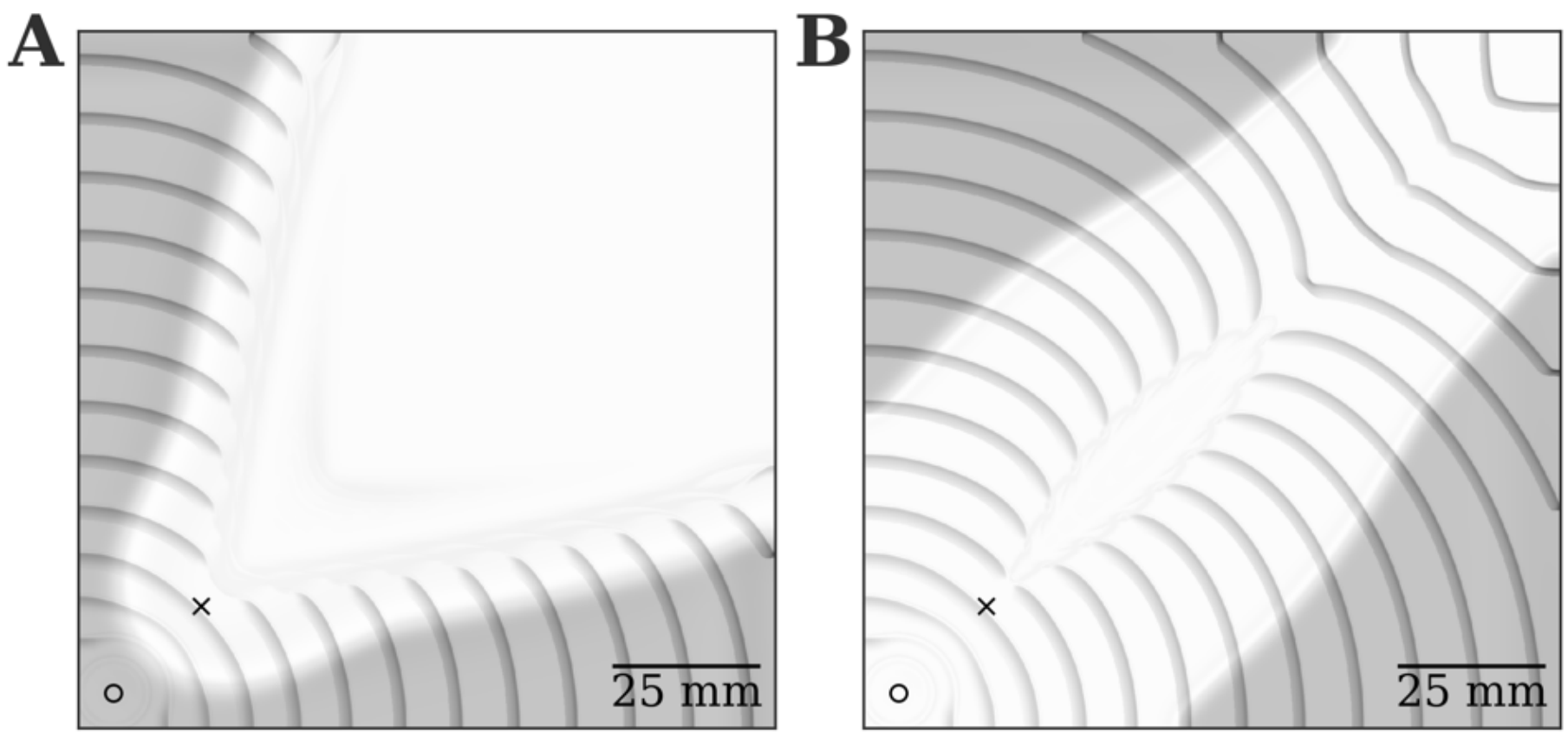

Supplementary Figure 4. Simulated dynamics of a T7 PhASER carried by *E. coli* W1485(F8) bacteria (host and phage inoculated at 'o' and 'x', respectively). In **A**, we simulate an exponentially distributed latent period given by $L = 1$, resulting in a phage infection propagation that outpaces bacteria chemotaxis as the lysis zone keeps expanding outwards (See 2-hour spaced chemotactic front positions overlaid in dark grey over final frame). In **B**, we decouple latent period and resources as $\eta(R) = \frac{\eta}{3}$, where the division by 3 ensures that the maximum lysis rate is the same as in the rest of T7 *vs E. coli* W1485(F8) simulations (see Supplementary Text Section 3). In this case phage infections at the very chemotactic front (overlaid in dark grey every 2 hours), where lysis rate is near its maximum, are transported as in the main figures. On the other hand, phage slowly consume the bacterial lawn behind the transport front, producing a constantly expanding lysis region that differs from the experimentally observed dynamics. All parameters are indicated in Supplementary Text Table S1.

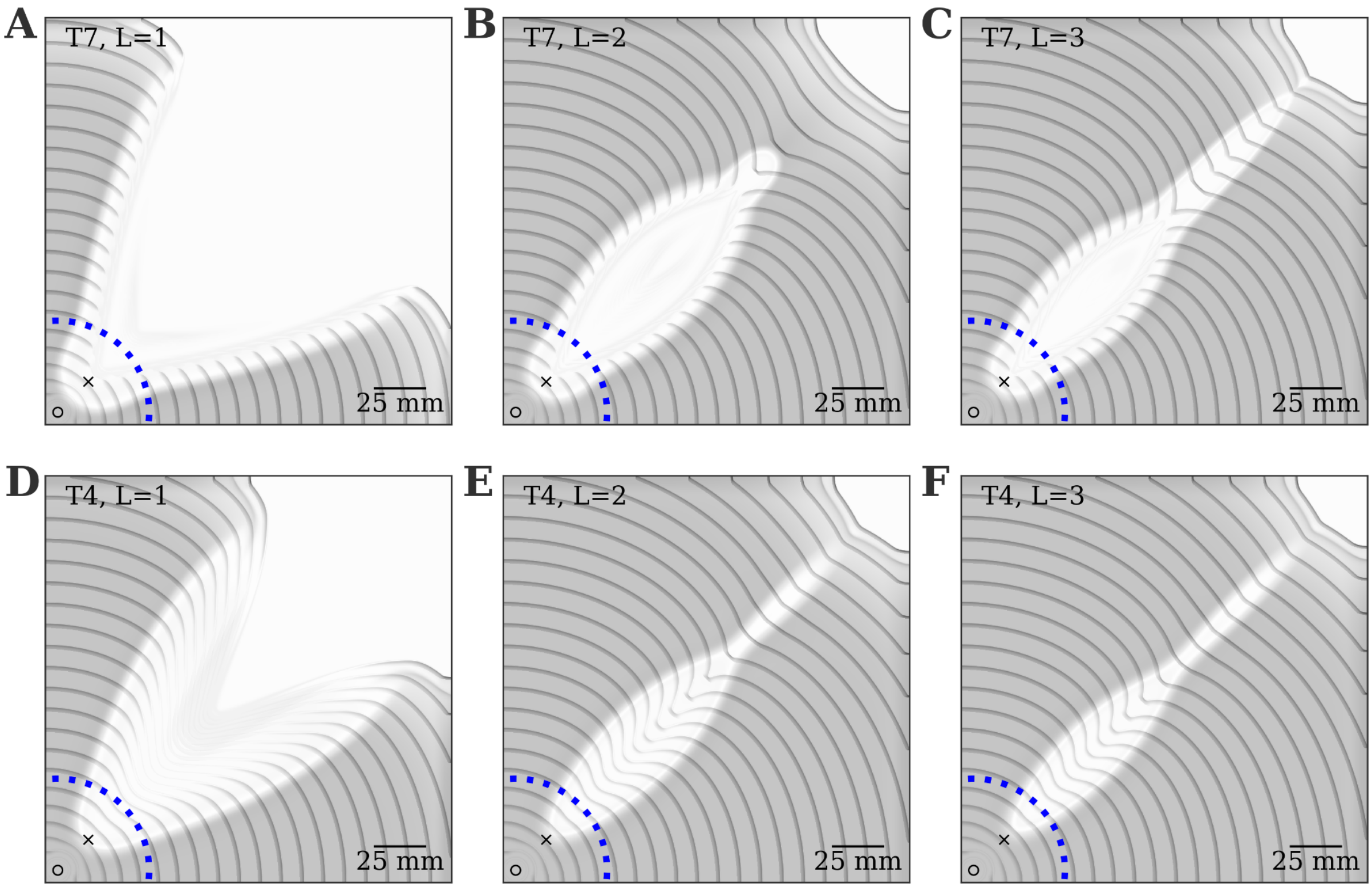

Supplementary Figure 5. Simulated dynamics of a PhASER carried by *E. coli* W1485(F8) bacteria (migrating from 'o'), for phages T7 **(A,B,C)** and T4 **(D,E,F)**, inoculated at 'x'. In **A** and **D** we simulate an exponentially distributed latent period (Coefficient of Variation CV=100%) given by $L = 1$, resulting in a phage infection that keeps pace with bacterial chemotaxis and generates tremendous lysis zones that reach the 24cm plate edge without reconverging. We then simulate the model dynamics while increasing L to 2, corresponding to a 70% CV **(B,E)**, and then to $L = 3$, corresponding to a 57% CV **(C,F)**. Final timepoint frames are overlaid with 2 hour-spaced chemotactic front positions (grey curves). Dotted blue lines outline the boundaries of a standard Petri dish with 9 cm diameter. All other parameters are indicated in Supplementary Text Table S1.

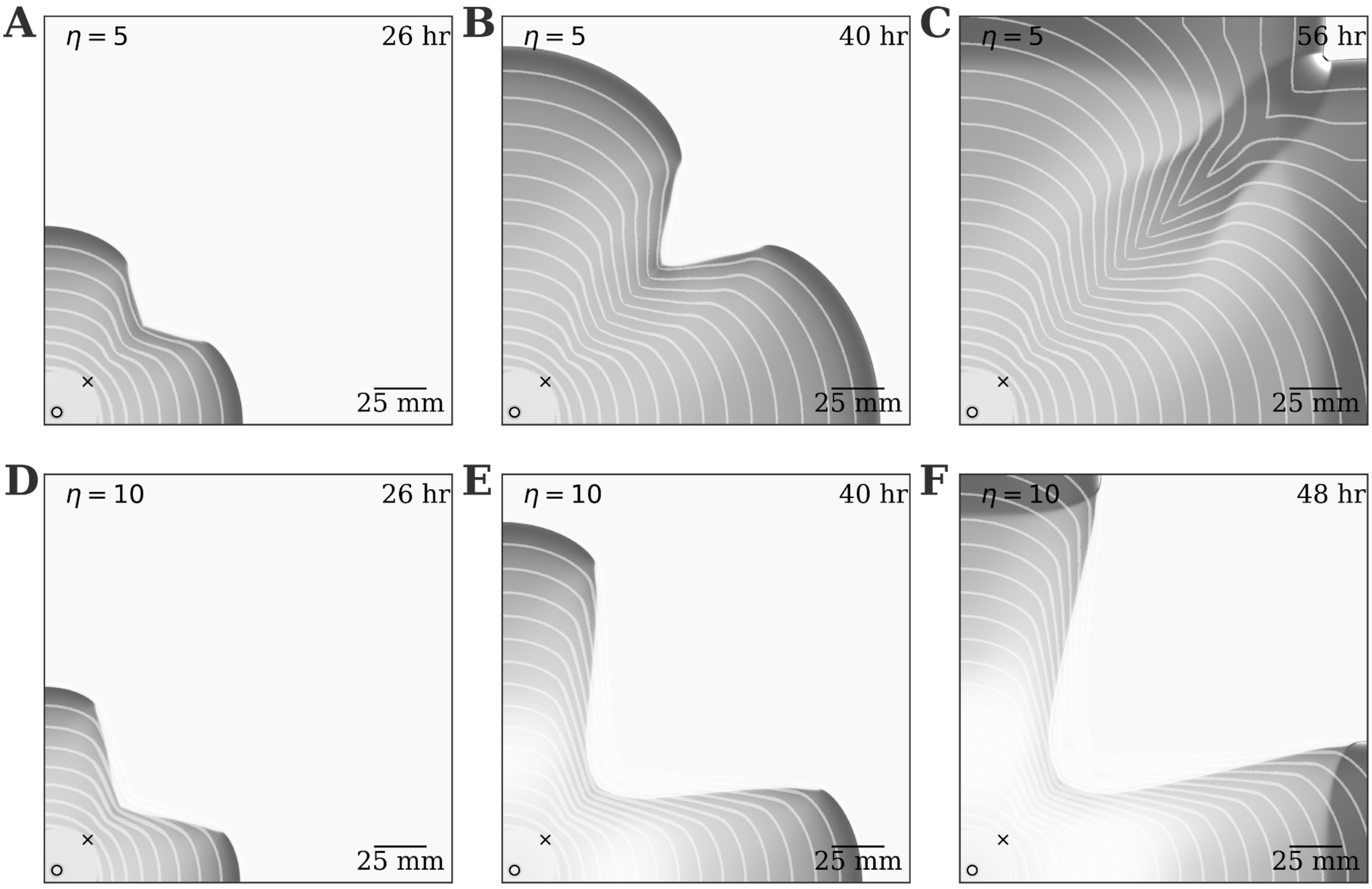

Supplementary Figure 6. Simulated dynamics of a T7 PhASER carried by *E. coli* W1485(F8) bacteria that track the main nutrient. χ is rescaled to $5 \cdot 10^7 \mu m^2$/hrs to keep the range expansion speed comparable with experiments. In **A,B,C** we keep the phage average lysis time fixed to the default T7 $\eta = 5$/hr, showing frames of the same simulation at 26, 40 and 56 hours respectively. In **D,E,F** we increased the lysis time to $\eta = 10$/hr to prevent invasion from the bacteria lawn for example purposes, showing frames of the same simulation at 26, 40 and 48 hours respectively. Final timepoint frames are overlaid with 2 hour-spaced front positions (white curves). All other parameters are indicated in Supplementary Text Table S1, except for a_- and a_+, here multiplied by K/K_2.

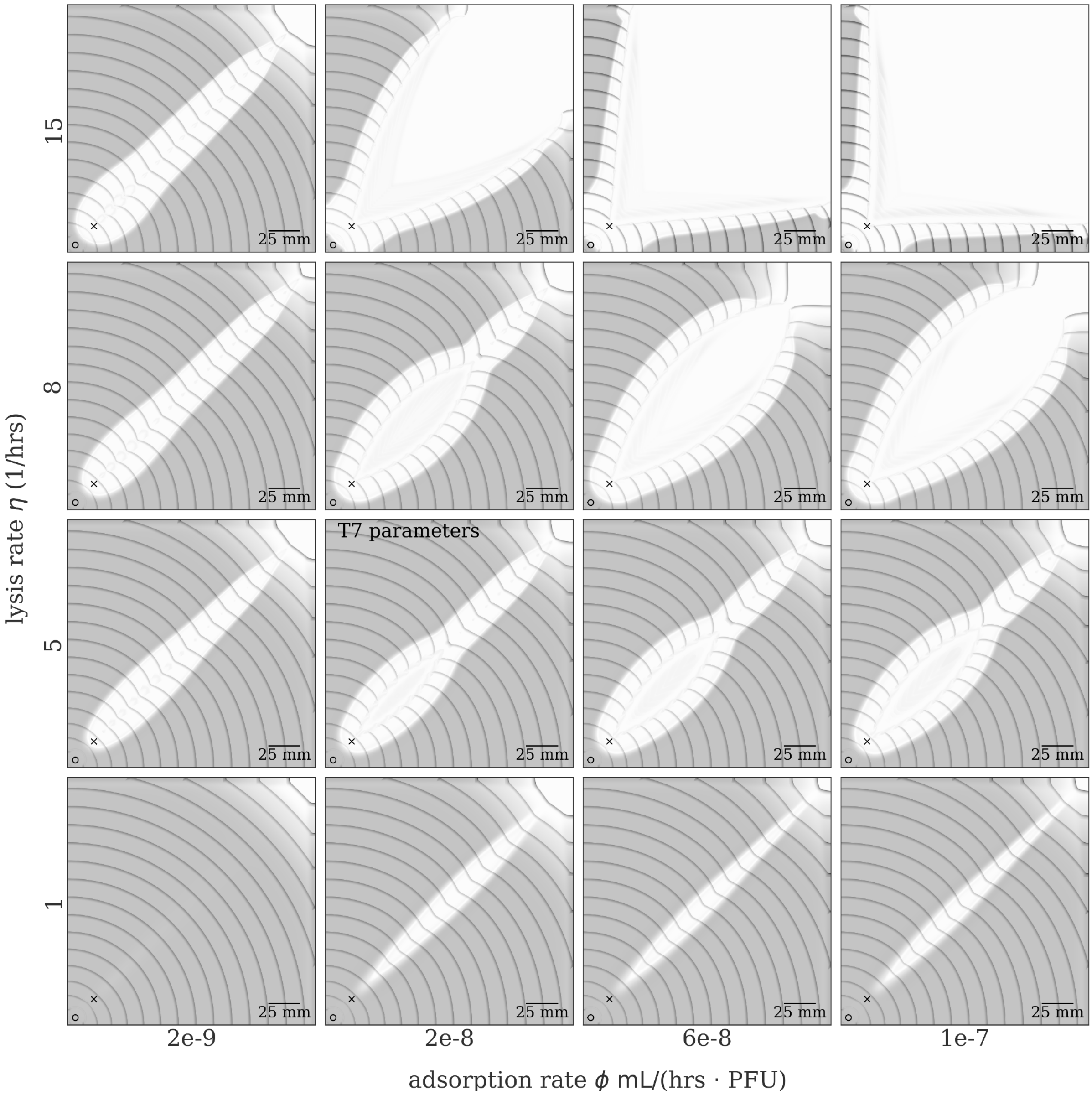

Supplementary Figure 7. Simulated dynamics of a PhASER carried by *E. coli* W1485(F9) bacteria when varying the lysis rate η between 1 and 15/hr, and the phage adsorption rate ϕ between $2 \cdot 10^{-9}$ and 10^{-7} mL/(hr PFU). PhASER emergence shows a remarkable robustness to 10-fold variations in viral life traits. For extremely low adsorption rates and lysis rates phage infections are unable to sustain PhASERs. Increasing either adsorption or lysis rates produce stable PhASERs with a wider and wider lysis region, until at very high values the chemotactic fronts at its sides do not reconverge within our experiment's spatial scales. Hosts and phage are inoculated at points ‘o’ and ‘x’, respectively. Final timepoint frames are overlaid with 2 hour-spaced front positions (grey curves). All other parameters correspond to those indicated for phage T7 in Supplementary Text Table S1.

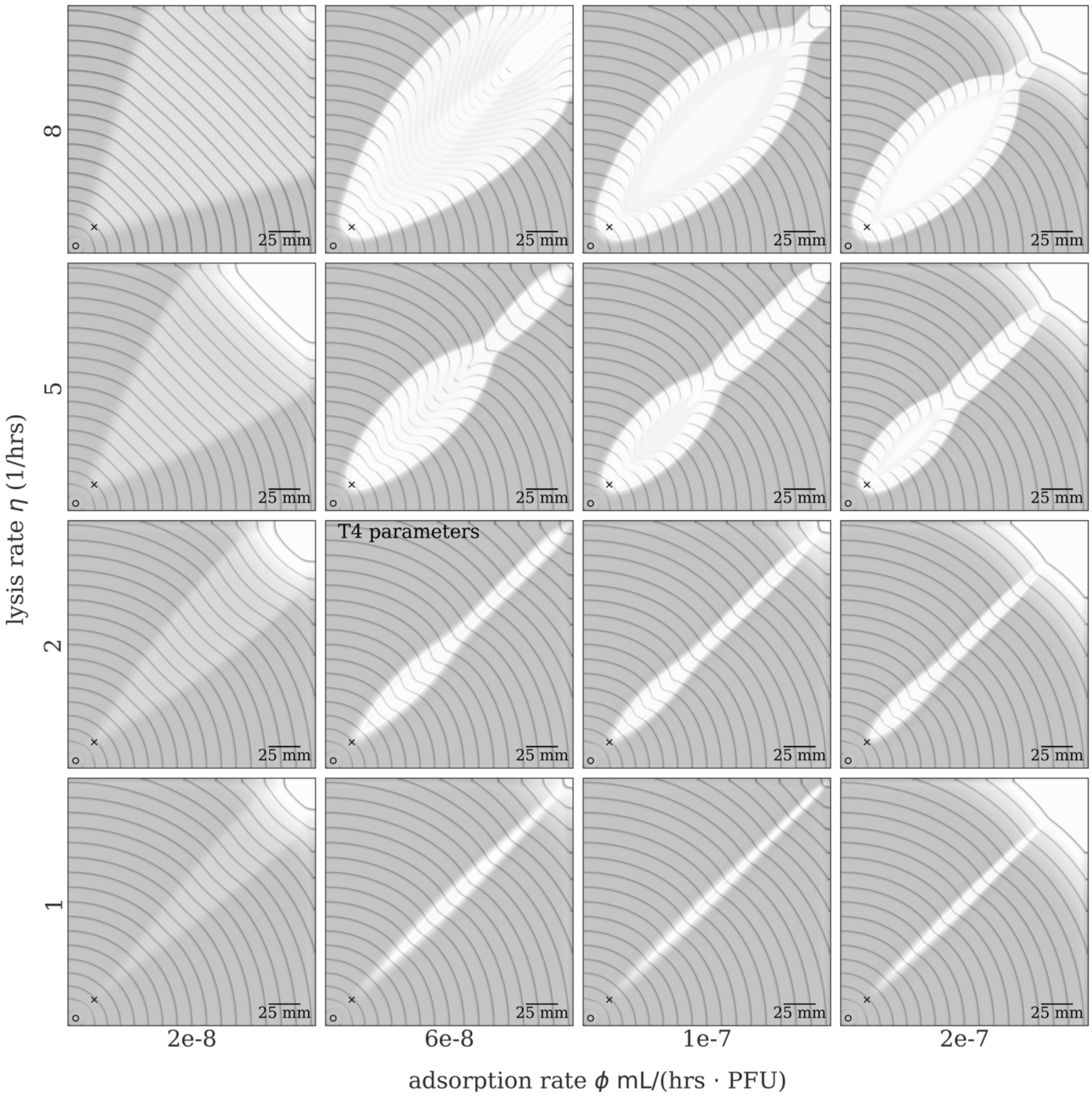

Supplementary Figure 8. Simulated dynamics of a PhASER carried by *E. coli* W1485(F9) bacteria when varying the lysis rate η between 1 and 8/hr, and the phage adsorption rate ϕ between $2 \cdot 10^{-8}$ and $2 \cdot 10^{-7}$ mL/(hr PFU), employing the T4 nonlinear saturation profile $F(P) = \phi \frac{P}{1+P/P_c}$ with $P_c = 10^7$ PFU/mL. PhASER emergence is robust variations in viral life traits spanning 1 order of magnitude. For extremely low adsorption rates phage infections do not drastically reduce bacteria densities at the center of the plate, and the vanishing curvature of the chemotactic front is not pronounced enough to produce the typical PhASER reconvergence within our experimental spatial scales. Increasing either adsorption or lysis rates produces stable narrow PhASERs with residual cells at its center, as observed experimentally for phage T4. Higher adsorption and lysis rates offset the nonlinear phage saturation profile and produce PhASERs that present T7-like features – *i.e.* initial curved fronts divergence and vanishing cell densities inside PhASERs. Hosts and phage are inoculated at points 'o' and 'x', respectively. Final timepoint frames are overlaid with 2 hour-spaced front positions (grey curves). All other parameters correspond to those indicated for phage T4 in Supplementary Text Table S1.

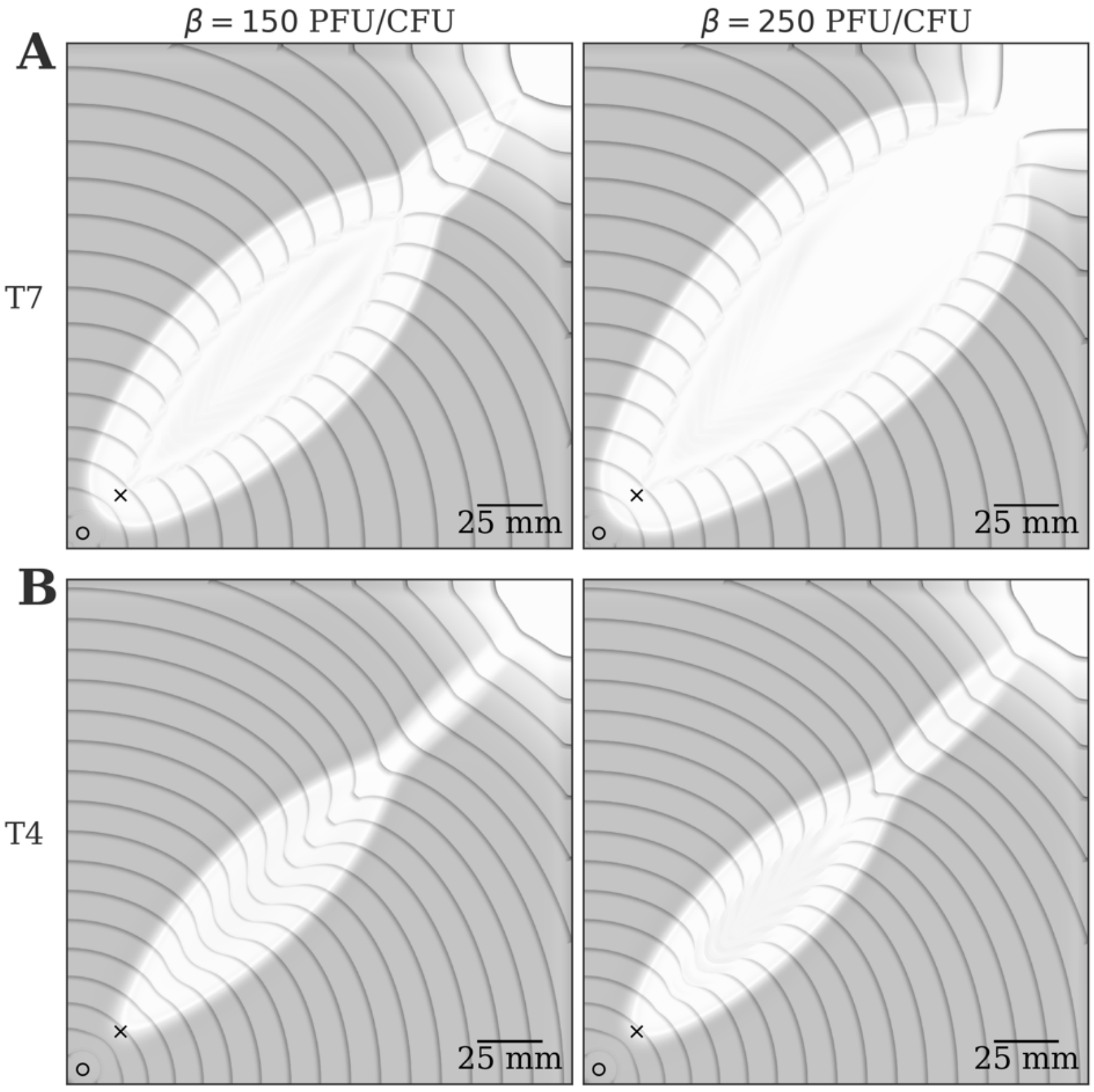

Supplementary Figure 9. Simulated dynamics of a PhASER carried by *E. coli* W1485(F9) bacteria when increasing the burst size β to 150 and 250 PFU/CFU, for phage T7 **(A)**, and T4 **(B)**. With $\beta = 150$ PFU/CFU we recover qualitatively similar PhASER dynamics as in Figure 3 in the main text. A higher $\beta = 250$ PFU/CFU yields T7 lysis regions that do not reconverge within our experiment's spatial scales, while T4 dynamics start presenting quantitative PhASER features typically associated with phage T7 – *i.e.* initial curved fronts divergence and vanishing cell densities inside PhASERs. Hosts and phage are inoculated at points ‘o’ and ‘x’, respectively. Final timepoint frames are overlaid with 2 hour-spaced front positions (grey curves). All other phage-dependent parameters correspond to those indicated in Supplementary Text Table S1.

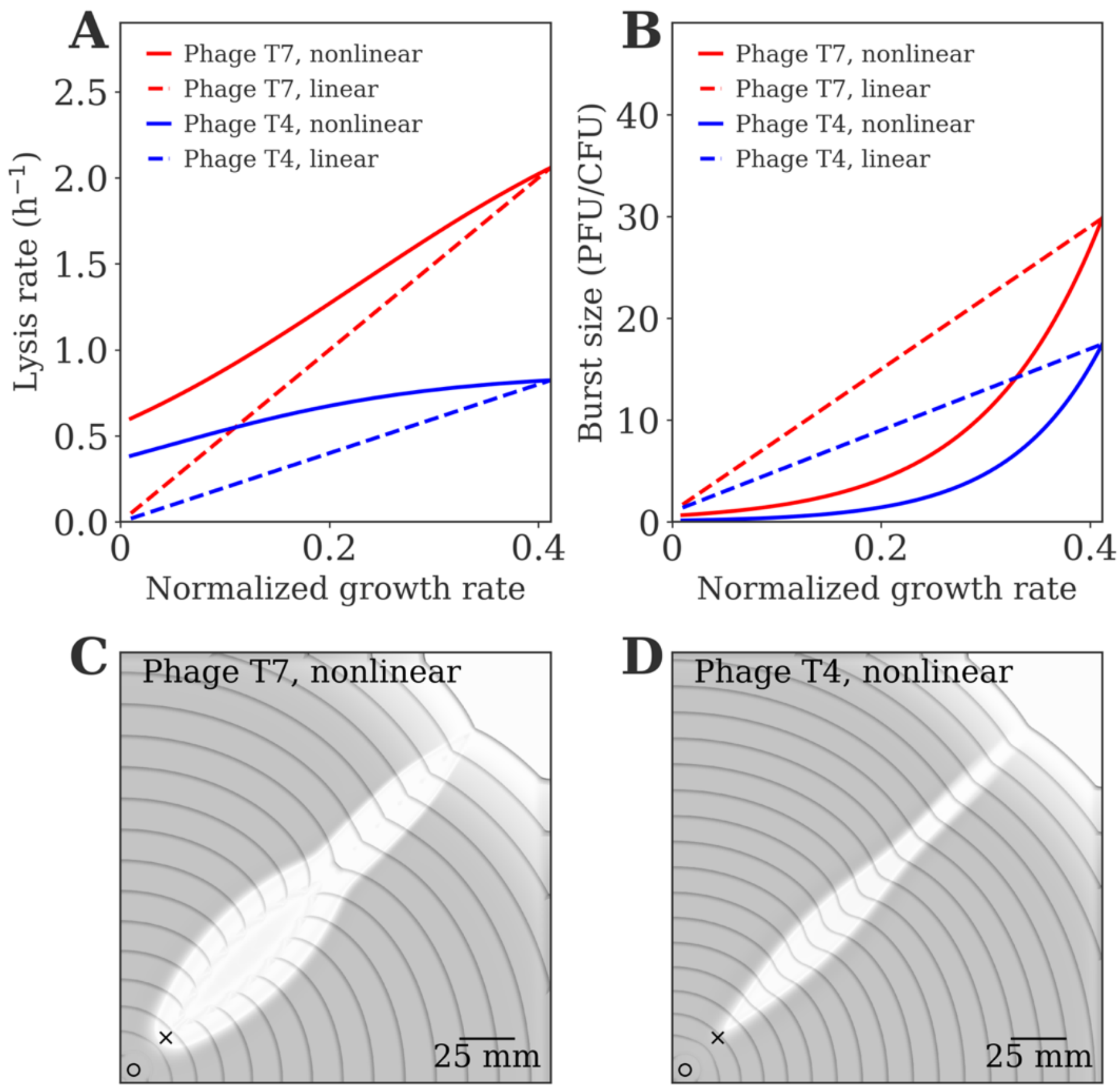

Supplementary Figure 10. Simulated dynamics of a PhASER model with nonlinear dependence of phage lysis rate and burst size with bacteria growth rate. Panels **A,B** compare the linear functions employed in the main text (dashed lines) for phage T7 (red) and T4 (blue), with the nonlinear lysis rate and burst size functions specified in equations S1 and S2 in the Supplementary text (full lines). These functions were adapted from empirically-informed phage traits dependence on hosts growth rate (*47*), details in the Supplementary Text. The x-axis spans the normalized bacteria growth rate range realized in the simulations based on the resources dynamics parameters – *i.e.* $\left[0, \frac{R_0}{R_0+K}\right]$, see Supplementary Text. Panels **C,D** show spatial simulations for phage T7 and T4 respectively with nonlinear phage traits, resulting in the formation of stable PhASERs. All other parameters are kept the same as in Figure 3 of the main text, as indicated in Table S1 for *E. coli* strain W1485(F9).

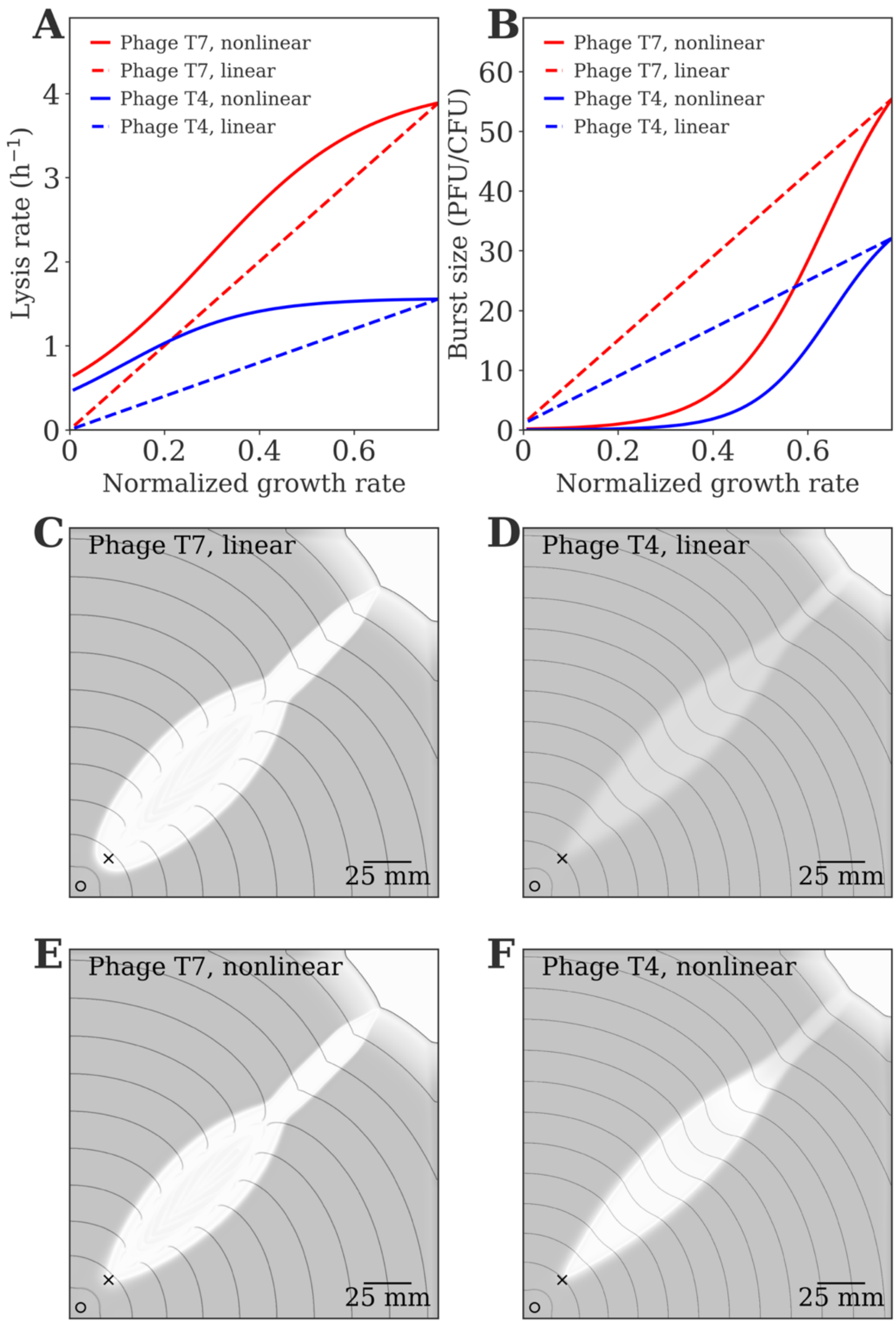

Supplementary Figure 11. Simulated dynamics of a PhASER model with a linear versus nonlinear dependence of phage lysis rate and burst size with bacteria growth rate, upon modifying the Monod constant parameter K from $5 \cdot 10^9$ to 10^9 CFU/mL – indicated in units of host cell concentrations as explained in the Supplementary text. Panels **A,B** compare the linear lysis rate and burst size functions (dashed lines) for phage T7 (red) and T4 (blue), with the nonlinear ones specified in equations S1 and S2 in the Supplementary Text (full lines). The x-axis spans the normalized bacteria growth rate range realized in the simulations based on the resources dynamics parameters – *i.e.* $\left[0, \frac{R_0}{R_0+K}\right]$, which is almost doubled compared to the baseline simulations, see Supplementary Text. Panels **C,D** show spatial simulations for phage T7 and T4 respectively with linear phage traits, while panels **E,F** show the model outcomes with the nonlinear functions plotted in **A,B**. Despite quantitative differences, all of these simulations resulted in the formation of stable PhASERs. All other parameters are kept the same as in Figure 3 of the main text, as indicated in Table S1 for *E. coli* strain W1485(F9).

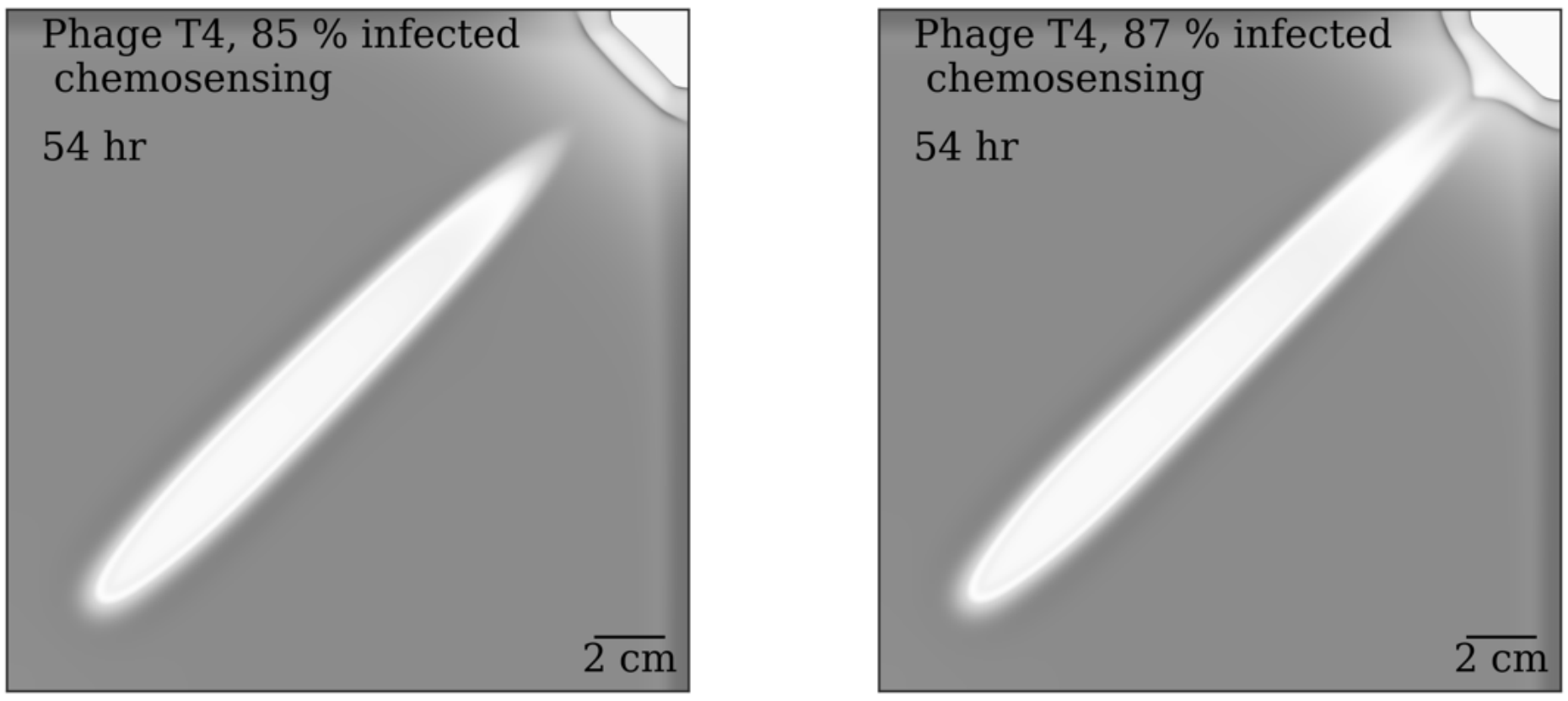

Supplementary Figure 12. Simulated dynamics of a T4 *vs E. coli* W1485(F8) PhASER model where the parameter governing infected cells' responsiveness to chemoattractant gradients is modulated as $\tilde{\chi} = \alpha\chi$ with α between 0 and 1. The stable PhASER propagation towards the top-right corner is lost when α is decreased from 0.87 to 0.85. All parameters are indicated in Supplementary Text Table S1.

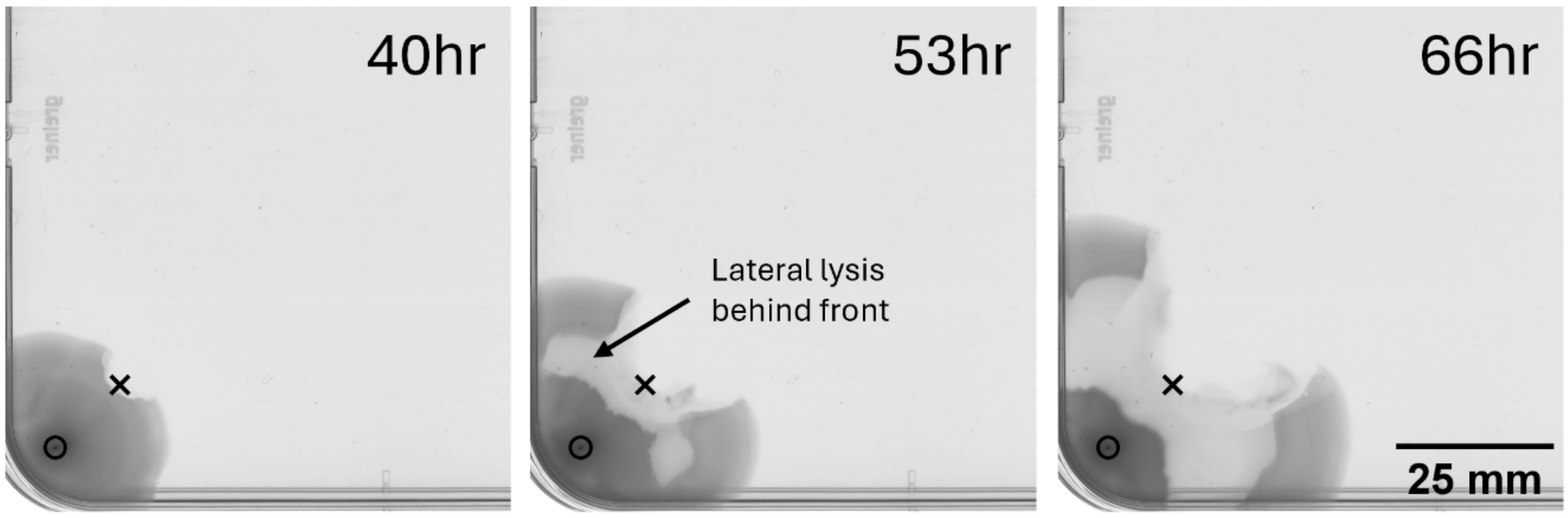

Supplementary Figure 13. Deviation from focused infection transport at low migration speeds in higher concentration swim agar. A colony (dark grey) of *E. coli* W1485(F8) grows and migrates at ~ 0.7mm/hr through an 0.4% agar LB swim assay from location ∘, encounters T7 phage at location ×, and displays a rapid lateral spread of lysis behind the migrating front. The timeseries is labelled with hours post inoculation.

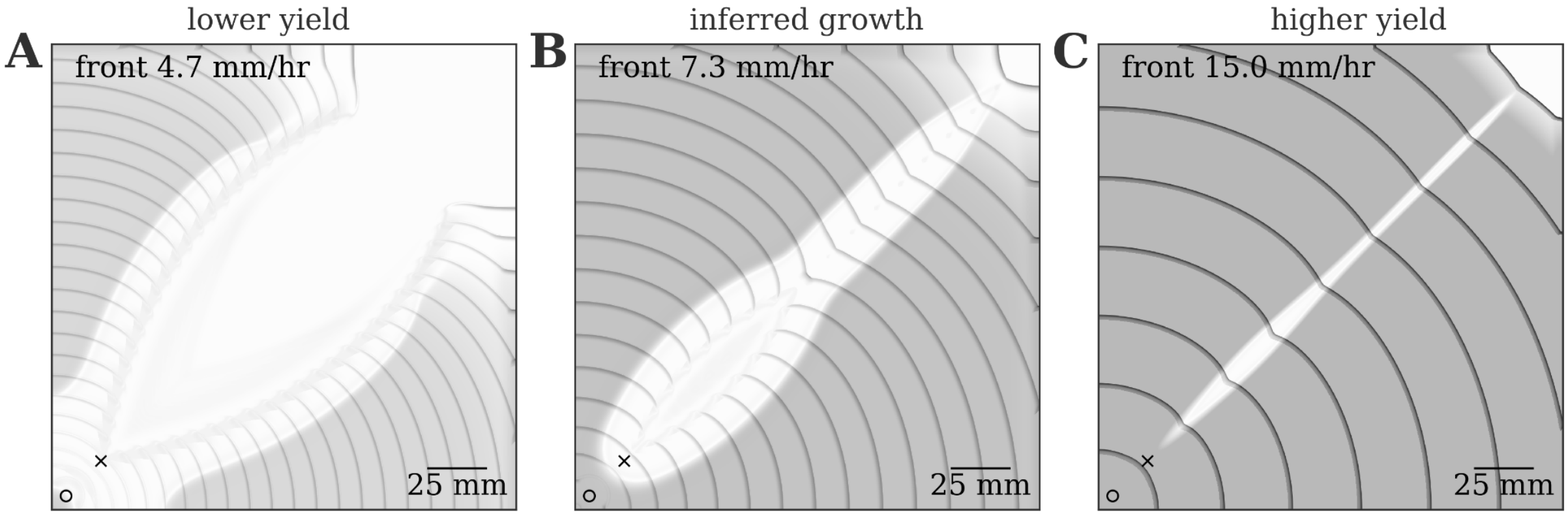

Supplementary Figure 14. Simulated dynamics of a PhASER carried by *E. coli* W1485(F9) bacteria (migrating from 'o'), for phage T7, inoculated at 'x'. In these simulations we modulated yield, *i.e.* the biomass conversion factor between nutrients and cells (as detailed in Supplementary Text), producing a growth rate either half **(A)** or four times **(C)** that originally inferred for this bacterial strain **(B)**. Note that, as expected, the population-mediated range expansion speed depends on the growth rate. Time frames are overlaid with 2 hour-spaced front positions (grey curves). All other parameters are indicated in Supplementary Text Table S1.

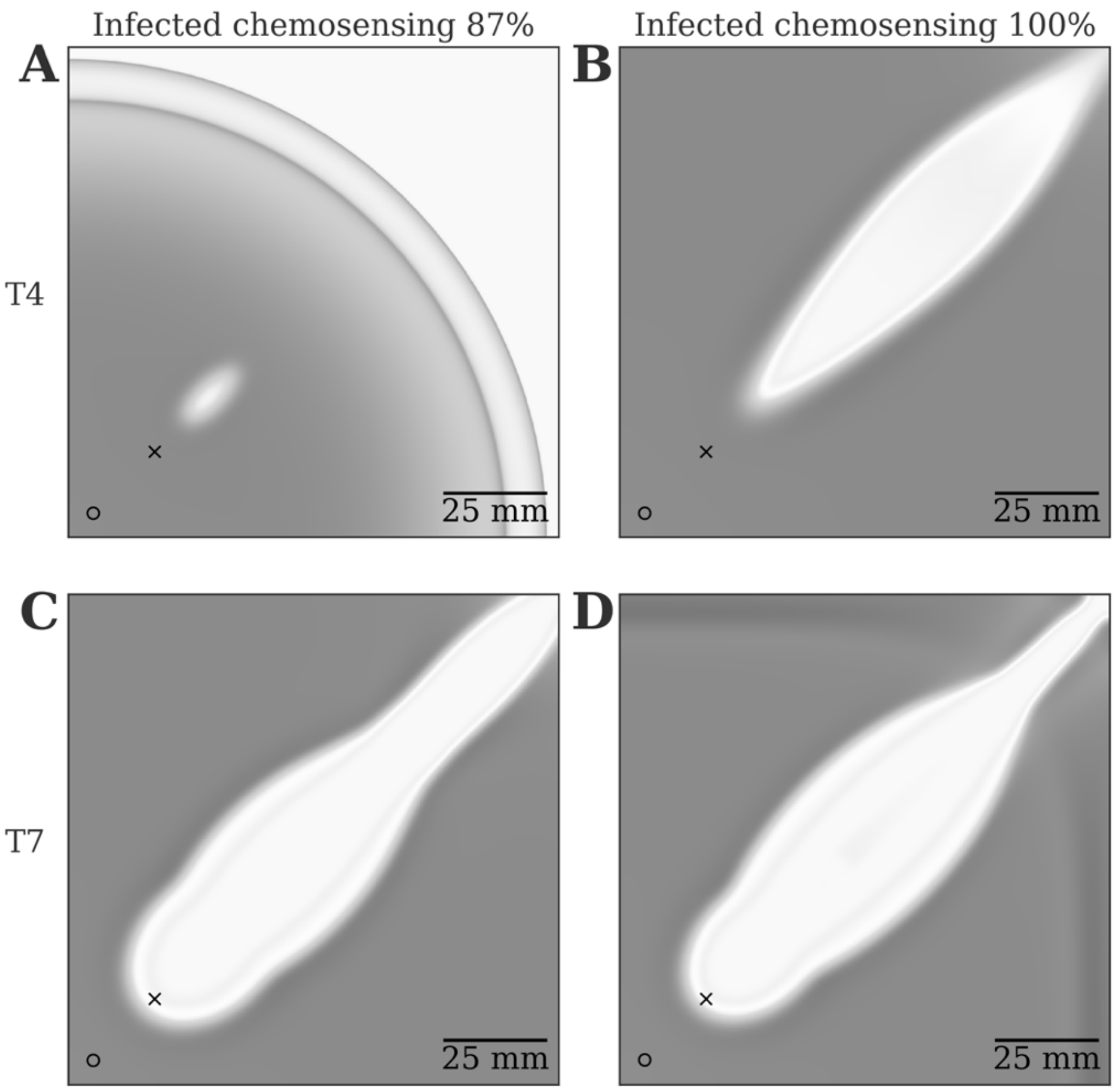

Supplementary Figure 15. Simulated dynamics of a T4 **(A,B)** and T7 **(C,D)** *vs E. coli* W1485(F8) PhASER model, where the parameter governing infected cells' responsiveness to chemoattractant gradients is modulated as $\tilde{\chi} = \alpha\chi$ with $\alpha = 0.87$ **(A,C)** and $\alpha = 1$ **(B,D)**. The simulations are initialized with a very low phage inoculum of $5 \cdot 10^5$ PFU/mL, leading to an estimated phage density of $4.7\ \Theta$ just before phage and bacteria come in contact. This choice corresponds to the phage inoculum in the second column of Figure 5E, with those two plots replotted here as panels **A** and **D** for comparison. For phage T4, reducing α leads to the dependence of stable PhASER propagation on phage inoculum density (**A** and Figure 5E), which is not realized with $\alpha = 1$ **(B)**. For phage T7, an equivalent reduction in α leads to a stable PhASER even at such low inoculum **(C)**, and therefore phage inoculum density does not affect PhASER formation like for $\alpha = 1$ (**D** and Figure 5E). All other phage-dependent parameters are indicated in Supplementary Text Table S1.

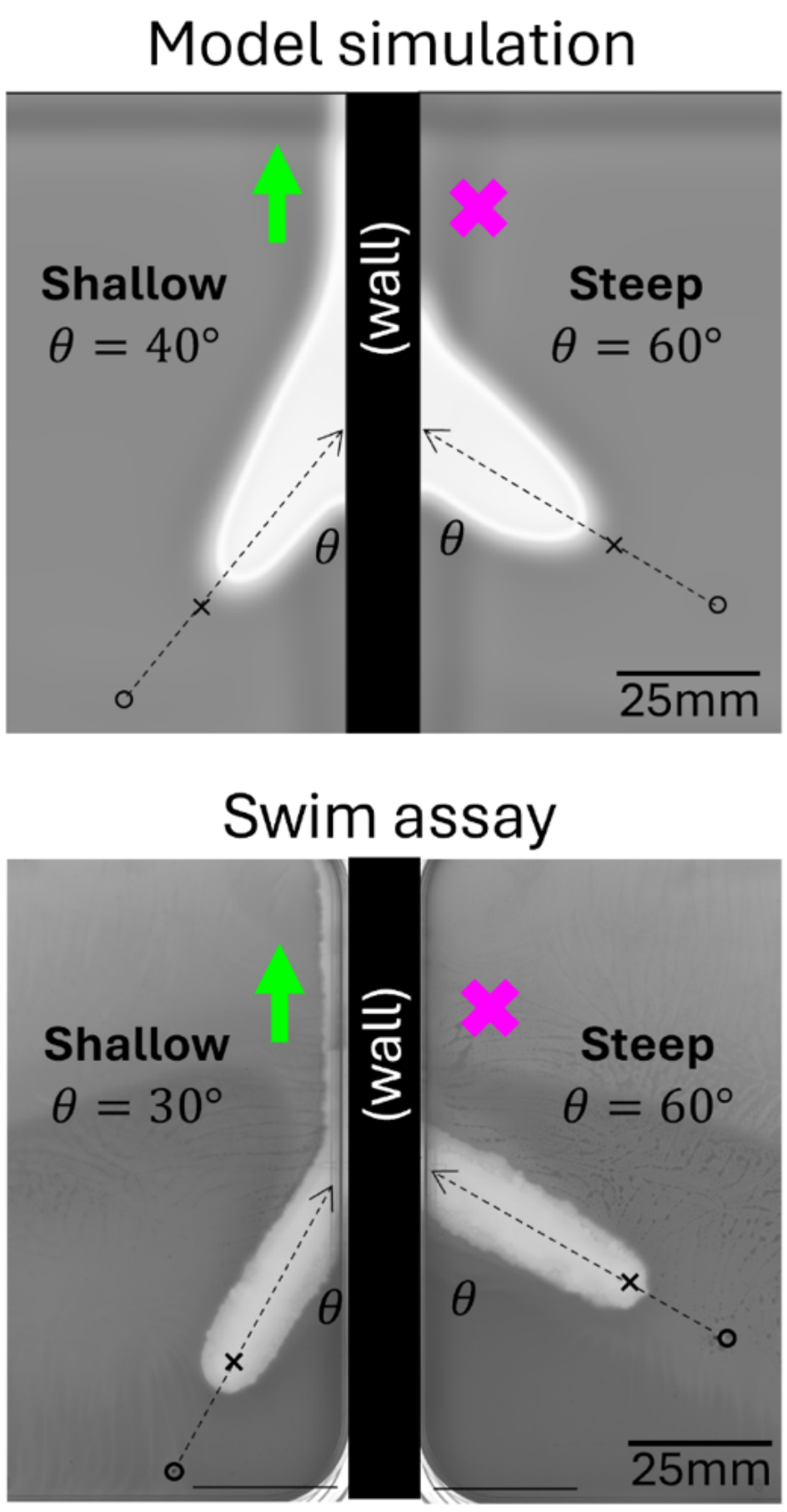

Supplementary Figure 16. PhASERs intersecting solid walls terminate or turn and propagate stably along them, depending on angle. Model simulations (top) and experimental swim assays (bottom) in which a bacterial host (*E. coli* W1485(F8) or parameters fit to same) grows and chemotaxes from an inoculation point ∘, to eventually cover a swim assay plate (dark grey growth). On encountering a spot of lytic phage (T4 or parameters fit to same) inoculated 20mm away at ×, a PhASER (cleared zone) forms in the migrating bacterial front and propagates straight (dashed arrow) until intersecting a solid wall. On the left, PhASERs intersecting the wall at shallow angles of roughly half that of the merging PhASERs in Figure 6A, in the simulation ($40°$) or swim assay ($30°$) turn and continue to propagate with the migrating host front along the wall (green arrows). PhASERs intersecting the wall at a steep angle (60°, half of merger angles in Figure 6B) lose the advancing front on collision and terminate (magenta crosses).

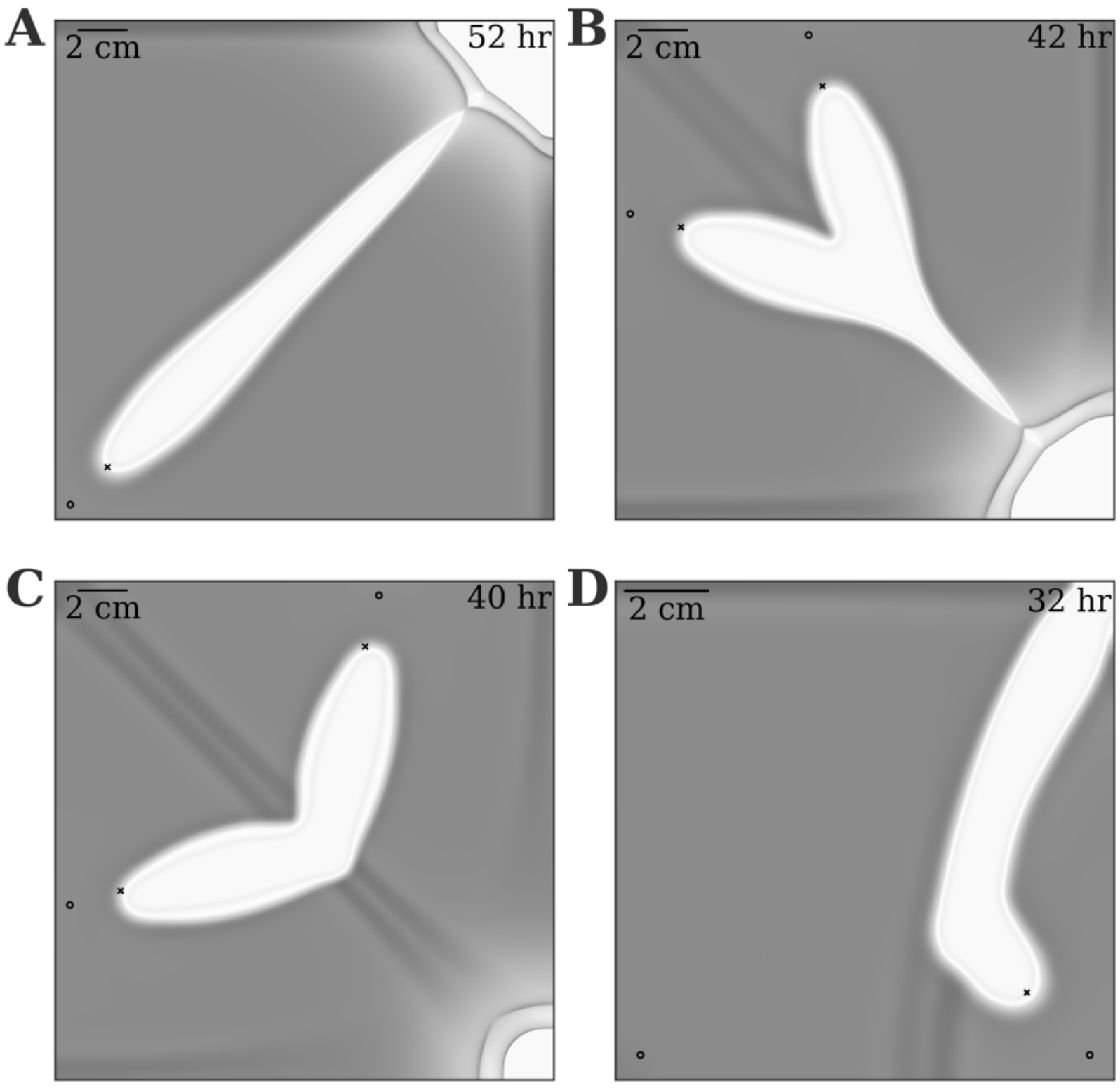

Supplementary Figure 17. Simulated dynamics of a T4 PhASER model with a linear adsorption profile $F(P) = \phi P$, transported by *E. coli* W1485(F8). The simpler adsorption profile maintains: **(A)** PhASER formation and stability, **(B)** robustness to collisions with other communities at 60 degrees , **(C)** PhASER termination upon collision at 120 degrees , and **(D)** stability to ecological perturbations challenging PhASERs with an *E. coli* W1485(F10) colony (inoculated at bottom-left) expanding at a much higher speed than W1485(F8) at bottom-right . Host and phage are inoculated at locations marked 'o' and 'x', respectively. All parameters are indicated in Supplementary Text Table S1, except for $\phi = 2 \cdot 10^{-9}$ mL/(hrs PFU), $\eta = 5$ hrs^{-1} and $\beta = 50$.

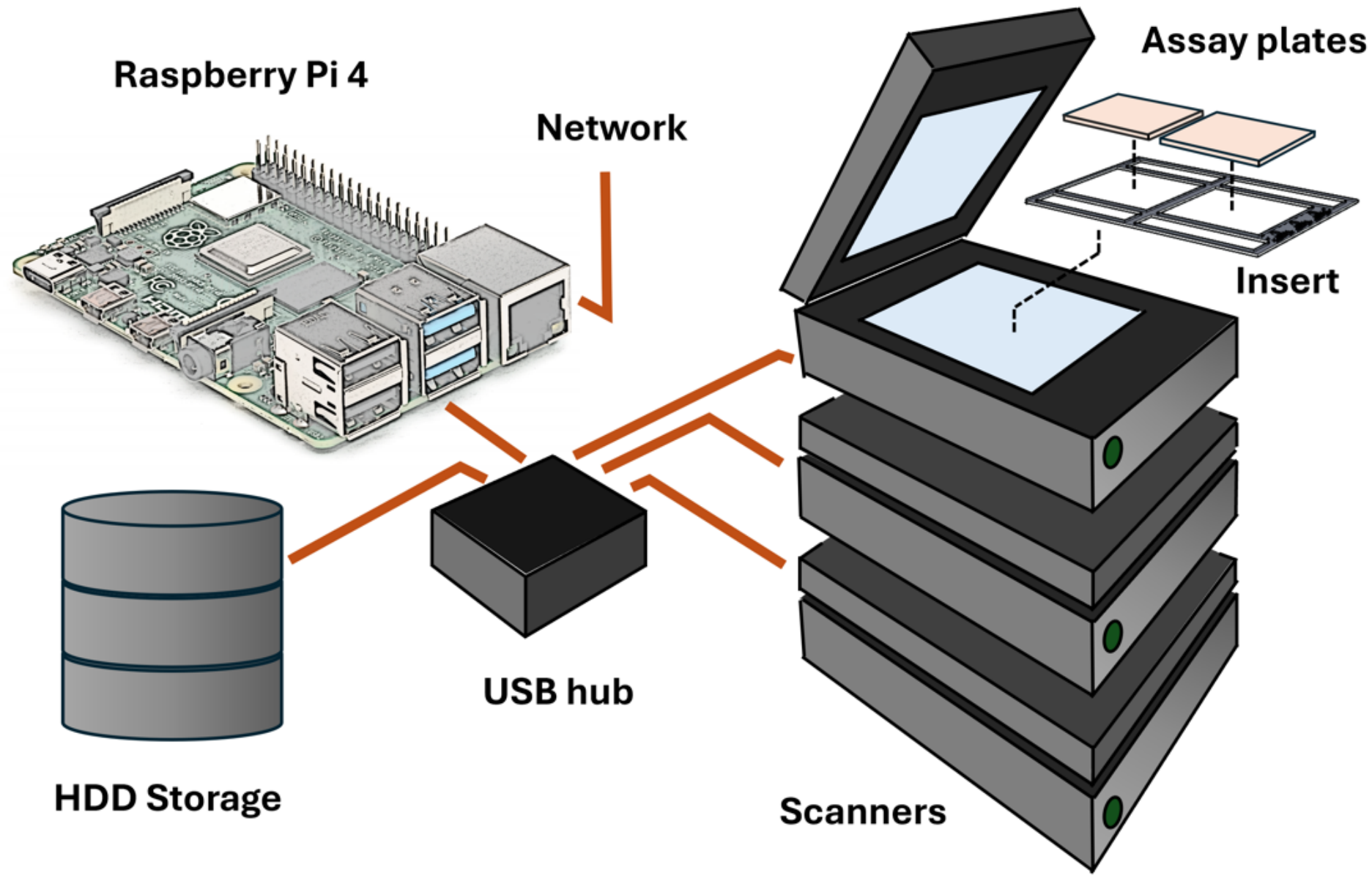

Supplementary Figure 18. Raspberry Pi-connected scanner cluster layout. Independent Raspberry Pi-controlled scanner timelapse modules are used to allow flexibility in running parallel experiments. Modules are composed of a Raspberry Pi 4 computer connected via USB hub to groups of (here, three Epson V800/850) scanners, and a local storage drive. The Raspberry Pi computers are run headless over the local network using VNC. A custom python script (Supplementary Method 1) is used to specify timelapse and image parameters and schedule scans and uses the SANE (http://www.sane-project.org/) interface to the scanners. Scanners are placed within temperature-controlled chambers. Custom 3D-printed inserts (Supplementary Method 2) are used to repeatably locate assay plates in the transmitted light imaging area of scanner platens.

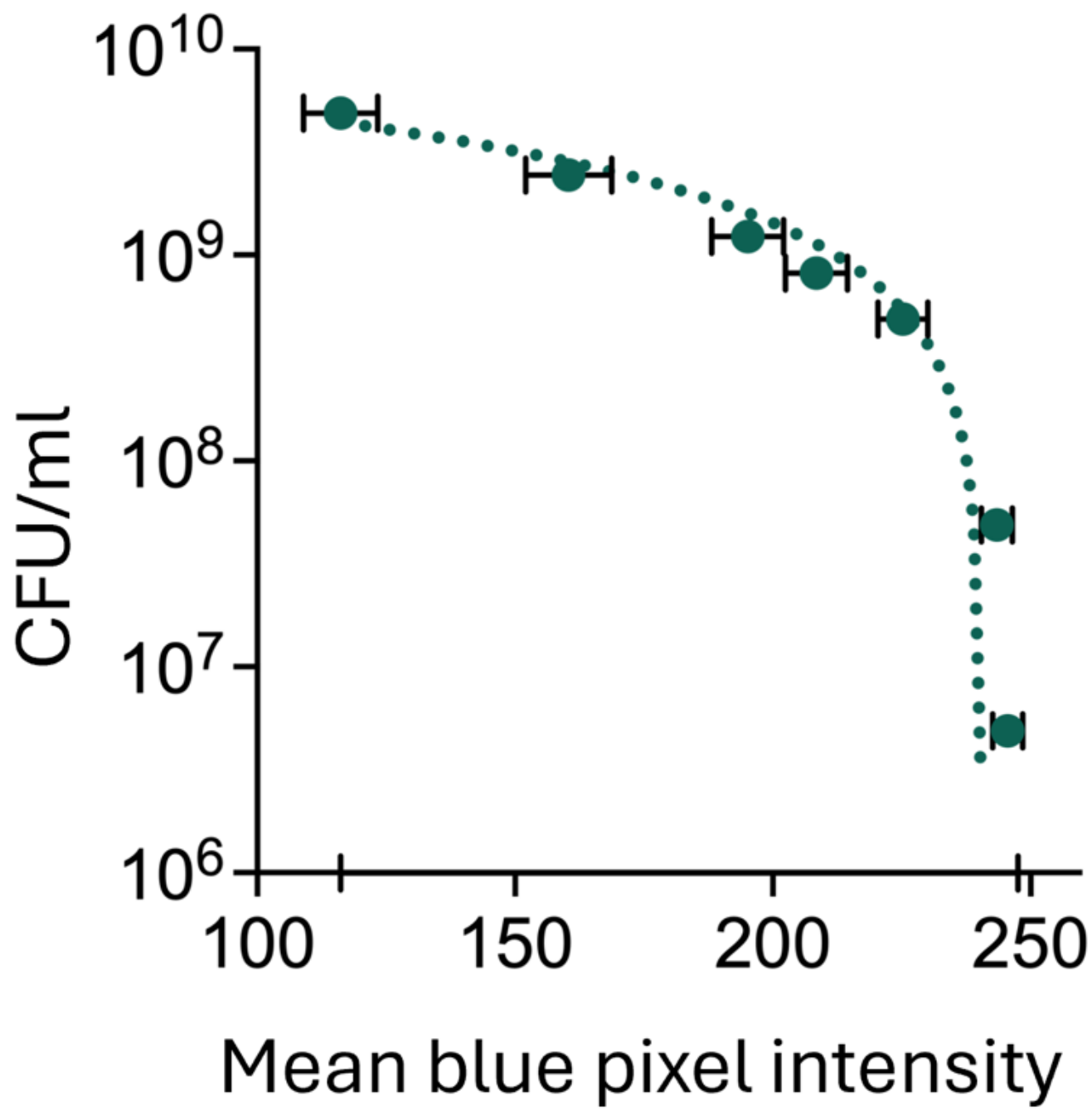

Supplementary Figure 19. Calibration curve (dashed, linear regression) used to map blue channel pixel intensity values to bacterial concentration (colony forming units (CFU) per ml) in scanned swim plate images (see Methods). 8-bit pixel intensities range from 0 to 255. Error bars represent standard deviation in pixel value across the plate area.

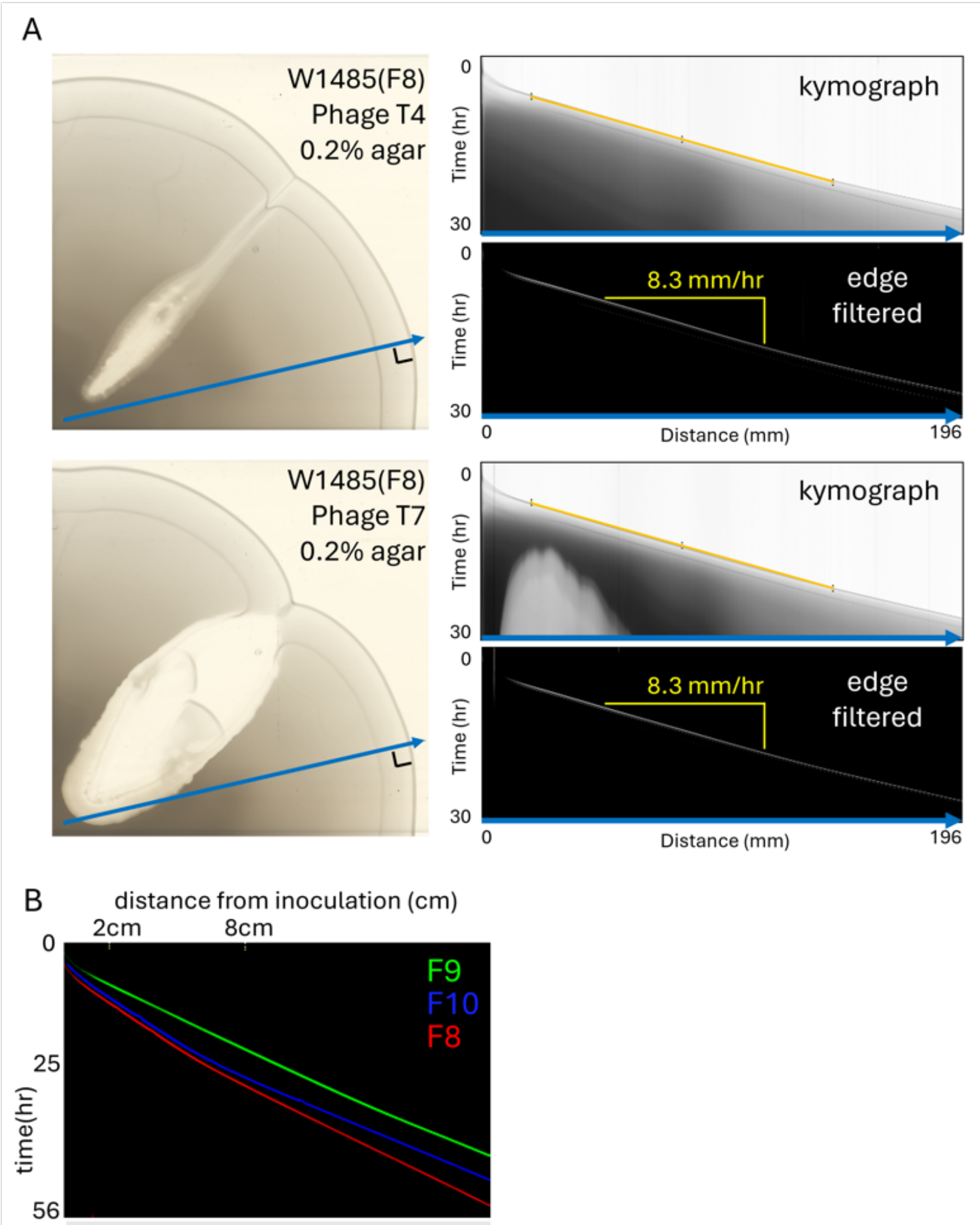

	2cm + 10hr	8cm + 10hr
W1485(F8)	5.5 mm/hr	6.9 mm/hr
W1485(F9)	7.1 mm/hr	7.4 mm/hr
W1485(F10)	5.8mm/hr	8.7 mm/hr

Supplementary Figure 20. Determining chemotactic range expansion rate using kymographs. **A.** Linear regions of interest (ROIs, blue arrows on scanned images of assay plates from Figure 5A) that are orthogonal to the migrating fronts throughout the timelapse sequence are specified, often extending from bacterial inoculation points. Kymographs, pixel intensity maps along the linear ROIs through time, are extracted from the full timelapse sequence (FIJI Multi Kymograph plugin) and displayed for the phage T4 (top) and T7 (bottom) assays. The orientation of the line profile is indicated by the blue arrow at the base (final timepoint) of the kymographs. Straight diagonal edges in these kymographs (overlaid by the orange line and highlighted using a sobel edge filter) indicate constant speed of advancement of the migrating front along the blue line. The reciprocal slope of this edge is the speed of the migrating front along the linear ROI. Speed during PhASER initiation is estimated by fitting front migration for 20 hours from phage inoculum contact. **B.** We observe that our *E. coli* assay strains with two chemotactic fronts (W1485(F8), W1485(F10)) tend to uniformly adjust to quicker migration speeds after ~20 hrs growth. Here, for migration in LB swim medium with 0.25% agar, the speed change is evident in the bent red and blue edge-filtered first migration fronts in the kymograph, and differences in each strain's measured front speed over the 10 hours after reaching 2cm and 8cm from inoculation. The speed adjustment is weaker in the W1485(F9) mutant strain which has a single dominant migration front (see straighter green front trajectory and more slight speed change).

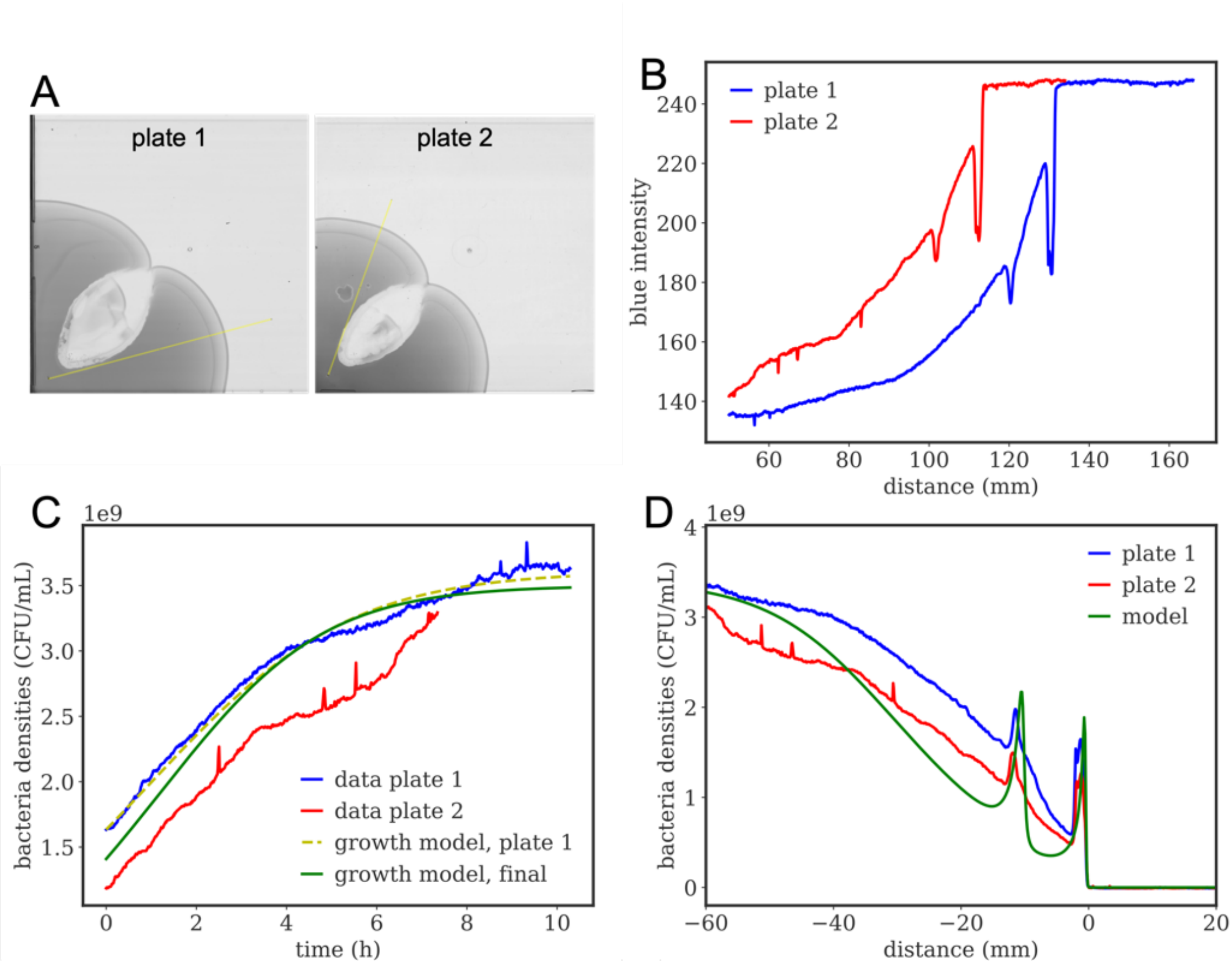

Supplementary Figure 21. Fitting pipeline to fix *E. coli* W1485(F8) bacteria life trait parameters. First, we select two plates with an expanding W1485(F8) colony, slicing the bacteria optical density along a radius parallel to the range expansion direction, in a region unaffected by phage lysis **(A)**. Then we record the blue channel intensity along the selected lines **(B)**, which is transformed to bacteria densities (CFU/mL) according to the calibration data in Supplementary Figure 9. Using the wave properties of the range expansion progressing at speed v, we transform the radial density profile measured a distance Δ behind the front – discarding the profile up to the last front if more than one is present – to growth that has been taking place for a time $t = \frac{\Delta}{v}$ since the front was at that location seeding cells behind it **(C)**. We use this indirectly derived growth curve to infer the parameters of a simple Monod growth model (see Supplementary Text Section 2 for details), where the yellow dashed line shows the growth model outcome with the parameters inferred on the plate 1 profile, while the green line shows the growth model dynamics with common parameters reproducing features from both plates (in this example, the fact that plate 2 initially has a higher growth rate, see Supplementary Text Section 2). Finally, we use these growth parameters in the full PDE model introduced in Box 1 to fit two parameters controlling chemical sensing and aspartate consumption rate matching the experimental radial density profile **(D)**. Bacteria responsiveness to sensed gradients is fixed to match v. Other W1485(F8) trait values are fixed from literature (see Supplementary Text Section 2).

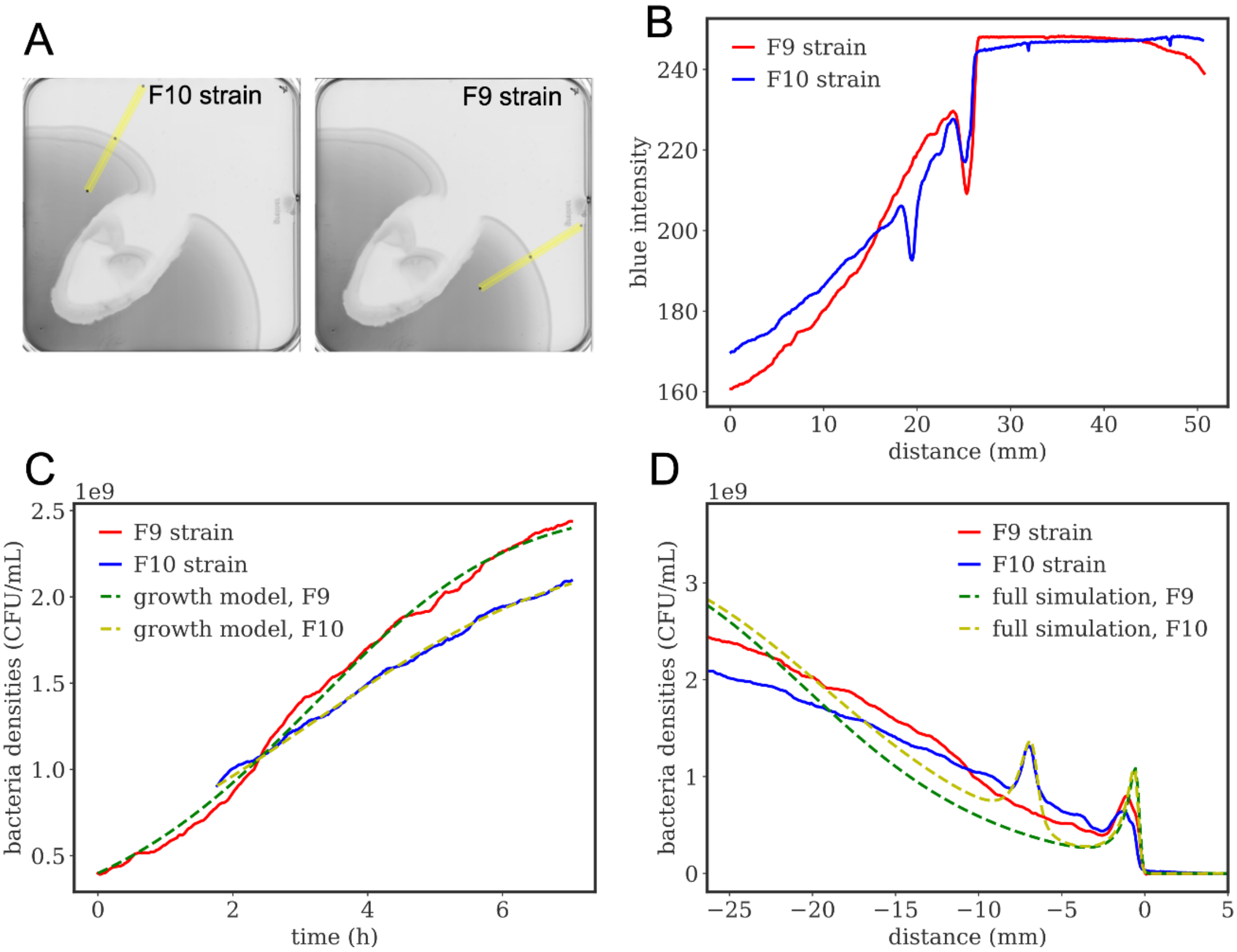

Supplementary Figure 22. Fitting pipeline to fix *E. coli* W1485(F9) and W1485(F10) strain life trait parameters. First, we select a plate with an expanding colony featuring *E. coli* W1485(F9) on the right of the plate and *E.coli* W1485(F10) on the left, slicing the bacteria optical density along a radius parallel to the range expansion direction, in a region unaffected by phage lysis **(A)**. For each strain we record the blue channel intensity along the selected lines **(B)**, which is transformed to bacteria densities (CFU/mL) according to the calibration data in Supplementary Figure 9. Then, using the wave properties of the range expansion progressing at speed v, we transform the radial density profile measured a distance Δ behind the front – discarding the profile up to the last front if more than one is present – to growth that has been taking place for a time $t = \frac{\Delta}{v}$ since the front was at that location seeding cells behind it **(C)**. We use this indirectly derived growth curve to infer the parameters of a simple Monod growth model (see Supplementary Text Section 2 for details), where the yellow dashed line shows the growth model outcome with the parameters inferred for strain W1485(F10) (blue), while the green dashed line shows the growth model dynamics with parameters inferred for strain W1485(F9). To avoid overfitting we choose common parameters for these two strains, compatible with experimental variation, to simulate the full PDE model introduced in Box 1 (details in Supplementary Text Section 2). Assuming the only difference between the two strains is W1485(F9) insensitivity to one of the two attractants, we fit a few sensing and uptake parameters (see Supplementary Text Section 2) to match the radial density profiles measured experimentally for each strain **(D)**.

Supplementary Text: Stable coexistence and transport of lytic phage infections with migrating bacterial hosts

S1. NUMERICAL IMPLEMENTATION

We simulate the Partial Differential Equation model introduced in Box 1 with a finite differences scheme. We introduce a 2D grid with 100 μm spacing, where population concentrations are tracked in time, mimicking the experimentally measured concentrations averaged in the vertical direction. We assign a virtual (*i.e.*, not explicitly simulated) height of 0.5cm to the grid, compatible with the soft agar layer in our experimental setup. The volume of each lattice site corresponds to $0.5 \cdot 10^{-4}$ mL. The size of the grid is either set to 12 or 20 cm, to match the corresponding agar plate format used in the experiments.

The spatial derivatives $\frac{\partial}{\partial x}, \frac{\partial}{\partial y}$ present in the diffusion and advection terms are discretized on the square lattice. We use the following stencil kernel to discretize the Laplacian operator ($\nabla^2 = \frac{\partial}{\partial x}^2 + \frac{\partial}{\partial y}^2$) in the diffusion terms:

$$\text{Kernel} = \frac{1}{\Delta x^2}\begin{pmatrix} 0.25 & 0.5 & 0.25 \\ 0.5 & -3 & 0.5 \\ 0.25 & 0.5 & 0.25 \end{pmatrix}$$

We convolve this kernel with the state fields. For instance, at time t, the diffusion term of resources $D_R\nabla^2 R$ at a site (x,y) can be obtained by discretizing spatial derivatives to first order with respect to first- and second-neighbor sites:

$$\begin{aligned}
D_R\nabla^2 R(t,\mathbf{x})|_{(x,y)} &= \frac{D_R}{\Delta x^2}\begin{pmatrix} 0.25 & 0.5 & 0.25 \\ 0.5 & -3 & 0.5 \\ 0.25 & 0.5 & 0.25 \end{pmatrix} * R(t,\mathbf{x})|_{(x,y)} \\
&= \frac{D_R}{\Delta x^2}\begin{pmatrix} 0.25 & 0.5 & 0.25 \\ 0.5 & -3 & 0.5 \\ 0.25 & 0.5 & 0.25 \end{pmatrix} \cdot \begin{pmatrix} R(t,x-\Delta x,y+\Delta y) & R(t,x,y+\Delta y) & R(t,x+\Delta x,y+\Delta y)) \\ R(t,x-\Delta x,y) & R(t,x,y) & R(t,x+\Delta x,y) \\ R(t,x-\Delta x,y-\Delta y) & R(t,x,y-\Delta y) & R(t,x+\Delta x,y-\Delta y) \end{pmatrix} \\
&= \frac{D_R}{\Delta x^2}\Bigg(-3R(t,x,y) + 0.5\Big(R(t,x+\Delta x,y) + R(t,x-\Delta x,y) + R(t,x,y+\Delta y) + R(t,x,y-\Delta y)\Big) + \\
&+ 0.25\Big(R(t,x+\Delta x,y+\Delta y) + R(t,x-\Delta x,y+\Delta y) + R(t,x+\Delta x,y-\Delta y + R(t,x-\Delta x,y-\Delta y)\Big)\Bigg),
\end{aligned}$$

where $*$ denotes a convolution operation while $\cdot$ denotes the inner product. Diffusion of viruses, chemoattractants and bacteria is given by the same equation accounting for their specific diffusion constant, noting that the bacteria diffusion term represents the random drift component of an active run-and tumble motion rather than passive Brownian motion.

The divergence of the velocity field determining chemotaxis takes the general form $\nabla \cdot f(\mathbf{x})\nabla g(\mathbf{x}) = \frac{\partial}{\partial x}\left[f(\mathbf{x})\frac{\partial}{\partial x}g(\mathbf{x})\right] + \frac{\partial}{\partial y}\left[f(\mathbf{x})\frac{\partial}{\partial y}g(\mathbf{x})\right]$ where the function f stands for the corresponding bacteria class density, while the function g stands for the chemosensing response $\chi \ln\left(\frac{1+a_n/a_-}{1+a_n/a_+}\right)$. We evaluate this differential operator on the discrete grid through a central differencing scheme. For example to evaluate $\frac{\partial}{\partial x}\left[f(\mathbf{x})\frac{\partial}{\partial x}g(\mathbf{x})\right]$ at a site (x,y), we interpolate f on the faces of the lattice site as $f(x-\frac{\Delta x}{2},y) = \frac{f(x,y)+f(x-\Delta x,y)}{2}$ and $f(x+\frac{\Delta x}{2},y) = \frac{f(x,y)+f(x+\Delta x,y)}{2}$. This allows us to evaluate the flux $f(\mathbf{x})\frac{\partial}{\partial x}g(\mathbf{x})$ at the site face $(x+\frac{\Delta x}{2},y)$ as $f(x+\frac{\Delta x}{2},y)\frac{g(x+\Delta x,y)-g(x,y)}{\Delta x}$. Finally, differentiating the fluxes on the site faces, we have:

$$\begin{aligned}
\frac{\partial}{\partial x}\left[f(\mathbf{x})\frac{\partial}{\partial x}g(\mathbf{x})\right]_{(x,y)} =& f(x+\frac{\Delta x}{2},y)\frac{g(x+\Delta x,y)-g(x,y)}{\Delta x^2} - f(x-\frac{\Delta x}{2},y)\frac{g(x,y)-g(x-\Delta x,y)}{\Delta x^2} \\
=& \frac{1}{2\Delta x^2}[f(x+\Delta x,y)g(x+\Delta x,y) + f(x-\Delta x,y)g(x-\Delta x,y) - 2f(x,y)g(x,y) \\
&+ f(x,y)g(x+\Delta x,y) + f(x,y)g(x-\Delta x,y) - f(x+\Delta x,y)g(x,y) - f(x-\Delta x,y)g(x,y)].
\end{aligned}$$

Generalizing to both dimensions through the whole grid we have:

$$\nabla \cdot f(\mathbf{x})\nabla g(\mathbf{x}) = \frac{1}{2\Delta x^2}\left[\begin{pmatrix} 0 & 1 & 0 \\ 1 & -4 & 1 \\ 0 & 1 & 0 \end{pmatrix} * f(\mathbf{x})g(\mathbf{x}) + f(\mathbf{x})\begin{pmatrix} 0 & 1 & 0 \\ 1 & 0 & 1 \\ 0 & 1 & 0 \end{pmatrix} * g(\mathbf{x}) - g(\mathbf{x})\begin{pmatrix} 0 & 1 & 0 \\ 1 & 0 & 1 \\ 0 & 1 & 0 \end{pmatrix} * f(\mathbf{x})\right].$$

When calculating spatial derivatives at the boundaries of the grid, we implement reflecting boundary conditions by adding a virtual site beyond the grid mirroring all population densities in the closest site of the real lattice, ensuring that all fluxes across the boundaries are 0. For example, at the right boundary $x = \Omega$, when calculating the convolutions with the diffusion and advection kernels for resources R, we set $R(t, \Omega + \Delta x, y) = R(t, \Omega, y)$. Together with the site-specific reaction terms, the spatial terms complete the computation of time derivatives given the state variables at a given time.

As the time derivatives are estimated, the system state is updated to the following time step through an explicit Euler scheme. The time step is changed adaptively throughout the simulation to increase the precision of the numerical integration. At each time iteration we check whether densities are updated to unfeasible negative values by fast reactions. In practice, we flag resource densities below $-K/1000$, attractant densities below $-3a_-$ and phage densities below $-\Theta/10$. If any of these conditions is met, the last iteration is disregarded and the time step is divided by 1.5 before repeating. Otherwise, negative concentrations are set to 0 and the system is updated to the next step, while the time step is multiplied by 1.3 up to a maximum of $5 \cdot 10^{-4}$ hr. The population densities are saved every 2 hours. When plotting the intensities profiles we convert CFU/mL to pixel intensities according to the calibration data in Supplementary Fig. 19.

Simulations are initialized with a constant level of resources and attractants, R_0 and a_0 respectively (specified and motivated below). Bacteria are initialized as a 2D Gaussian profile centered around the initial position $(x, y) = (6, 6)$mm, where the maximum density is set to $8 \cdot 10^8$ CFU/mL. The standard deviation of the initial profile is set to 2 mm. Similarly, phage are initialized as a Gaussian profile centered around $(21, 21)$mm with standard deviation 0.2mm and maximum value 10^9 PFU/mL. These initial conditions ensure that the peaks of bacteria and viruses distributions are at $1.5\sqrt{2} \sim 2$ cm similar to the inoculi distance in the experiments. At the beginning of the simulation there are no infected cells. The only simulations with different initial conditions are those shown in Figure 5E varying phage inoculum. Each column therein, from left to right, corresponds to an initial phage density of $5 \cdot 10^4$, $5 \cdot 10^5$, $7 \cdot 10^5$, 10^6 and $5 \cdot 10^6$ PFU/mL. The corresponding PFU count reported in the figure, expressed in units of the discreteness density threshold Θ, is estimated right before the range expansion front encounters the phage inoculum.

The source code is available at `https://github.com/Jacopo-Marchi/PhASER_PDEs`.

S2. PARAMETER ESTIMATION

In this section we detail the procedures we employed to fit parameters to reproduce our data or to qualitatively estimate them based on first principles, and we specify when we adopt parameters inferred in similar conditions in previous works. The resulting parameter values, biological meaning, and sources (when applicable) are reported in Table S1. When fitting a parameter to our experiments yielded a value comparable to a previously reported one, the "Source" column indicates the reference to the published value after the mention "Fitted". We stress here that most of the model parameters are effective parameters driving population dynamics in our phenomenological model. Whenever possible we constrained our parameter choices on studies inferring mechanistic quantities from microscale measurements such as (*36*, *54*, *73*, *74*, *77*). We clarify here that the qualitative dynamics presented in the main text are robust to variation in many of the fitted model parameters that do not find a counterpart in literature (see Section S3)– *i.e.* those without a citation on the "Source" column of Table S1. Therefore PhASER formation is not contingent on the details of the parameters estimation procedure presented in this Section. Instead, we used the parameters estimation procedure detailed below to capture quantitative features of our experimental measurements.

- Bacteria growth

  In order to fit bacteria life traits parameters, we consider experiments for each strain taking only into account phage-free portions of the swim assay, away from the lysis region. We take the radial intensity profile measured on a 0.25% agar plate inoculated with *E. coli* W1485(F8), far from phage T7 action (see Supplementary Fig. 21A), a proxy for bacteria density in the direction of range expansion. We normalize the intensity value to remove the artificial signal due to agar and scanner auto-calibration (Supplementary Fig. 21B). To do so we rescale the maximum intensity measured in the empty portion of the plate to the control intensity value measured in liquid medium without any bacteria in Supplementary Fig. 19. Then, we transform intensities to bacteria densities based on a spline interpolated on the intensity to CFU calibration data, as in Supplementary Fig. 19. First, we seek to infer the parameters related to bacterial growth, namely the initial amount of resources R_0, controlling the maximum bacterial density and the parameters controlling the bacterial growth profile, namely K and r. The chemotactically migrating front seeds cells that start growing behind it (*36*). We measure the speed v at which the front is moving when the profile is recorded, allowing us to deduce that bacteria growing at a distance Δ behind the front have been seeded there at a previous time $t_s = \Delta/v$. Hence, the transformation $t = (r_{peak} - r)/v$

Model parameters					
Variable	*Meaning*	*Value for different strains*		*Units*	*Source*
		E. coli W1485:			
		(F8)	(F9) and (F10) (if different)		
r	Maximum bacteria growth rate	1.3	1.6	hrs^{-1}	Fitted
K	Growth Monod constant	$7 \cdot 10^9$	$5 \cdot 10^9$	CFU/mL	Fitted
K_1	Aspartate Monod constant	3.2	1.5	μg/mL	Fitted
K_2	Serine Monod constant	10	3	μg/mL	(*39*, *72*), adjusted for mutants
$\mu_{1,\max}$	Aspartate uptake rate	$2 \cdot 10^{-8}$	$3 \cdot 10^{-8}$	μg/(cell × hrs)	(*73*), adjusted for mutants
$\mu_{2,\max}$	Serine uptake rate	$1.4 \cdot 10^{-7}$	-	μg/(cell × hrs)	(*73*)
a_-	Low chemosensing constant	0.3	-	μg/mL	(*39*, *54*, *74*)
a_+	High chemosensing constant	10	100	μg/mL	Fitted, also (*36*, *39*)
D_B	Bacteria diffusion constant	$1.8 \cdot 10^5$	-	μm^2/hrs	(*36*)
χ	Chemosensing responsiveness	$6 \cdot 10^6$	$4 \cdot 10^6$	μm^2/hrs	Fitted
S_0	Initial cell density	$8 \cdot 10^8$	-	CFU/mL	Experimental protocol
		Phage:			
		T7	T4		
ϕ	Infection rate	$2 \cdot 10^{-8}$	$6 \cdot 10^{-8}$	mL/(hrs · PFU)	Fitted
η	Maximum lysis rate	5	2	hrs^{-1}	Fitted
β	Burst size	70	40	PFU/CFU	Fitted
ω	Viral decay	0.01	-	hrs^{-1}	(*75*)
D_P	Phage diffusion constant	10^4	-	μm^2/hrs	(*76*)
L	Number of infected states	10	-		(*39*, *41*)
V_0	Initial viral density	10^9	-	PFU/mL	Experimental protocol
		Chemicals properties:			
D_R	Chemicals diffusion constant	$3 \cdot 10^6$	-	μm^2/hrs	(*77*, *78*)
R_0	Initial resource level	$3.5 \cdot 10^9$	-	CFU/mL	Fitted, also (*36*, *39*)
a_0	Initial chemoattractants level	20	-	μg/mL	(*36*, *39*)

TABLE S1: **Model parameters.** This table lists the baseline parameters used to simulate all strains. The values listed here for a given strain of bacteria or phage are used for all simulations featuring that strain, unless noted otherwise. The only parameters that are modulated from this baseline are D_B and χ to reflect variations in range expansion speed given changes in the agar concentration, and the values reported here refer to the standard condition 0.25% agar. Notably, in order to avoid overfitting, phage infection parameters are consistently used across all simulations of different cell strains, although in general phage traits could depend on the host type if bacteria chemotaxis properties were pleiotropically linked to susceptibility. The procedure to fit or qualitatively estimate parameters, as well as the conversions between different units used in other works, is detailed below.

allows us to obtain the growth curve, *i.e.* bacteria densities as a function of time after seeding, from the spatial radial profile. We discard the part of the curve corresponding to the radial profile until the second chemotaxis front to avoid effects driven by bacteria transport rather than growth. We infer R_0, r and K by minimizing the squares of the residuals between this data and a Monod growth model equivalent to the growth terms in Box 1, namely:

$$\frac{\partial R}{\partial t} = -r\frac{R}{R+K}S$$
$$\frac{\partial S}{\partial t} = r\frac{R}{R+K}S$$

where resources are expressed in units of cells, and bacteria start from a density S_0 fixed to the first data point in the curve. The optimization algorithm (*79*), applying the Levenberg-Marquardt method (*80*), yields $r = 1.1$ hr^{-1} with an estimated standard error of 0.2 hr^{-1}, $K = 8 \pm 1.6 \cdot 10^9$ CFU/mL, and $R_0 = 2 \pm 0.1 \cdot 10^9$ CFU/mL to which we need to add the bacteria density after the second peak $S_0 = 1.6 \cdot 10^9$ to retrieve the corrected final bacteria density when starting from a small amount of cells, leading to $R_0 = 3.6 \pm 0.1 \cdot 10^9$ CFU/mL. To account for variability in our data we repeat this procedure on another 0.25% agar plate inoculated with *E. coli*

W1485(F8), yielding $r = 1.3 \pm 0.7$ hr^{-1}, $K = 8 \pm 0.3 \cdot 10^9$ CFU/mL and $R_0 = 3.2 \pm 0.05 \cdot 10^9$ CFU/mL. We obtain estimates on the same order of magnitude, despite the fact that the preparation of the soft agar drives a certain degree of variability across experimental replicates. As final parameter estimates we select $R_0 = 3.5 \cdot 10^9$ CFU/mL, $K = 2R_0 = 7 \cdot 10^9$ CFU/mL and $r = 1.3$ hr^{-1}, compatible with the values inferred from both replicates. Supplementary Fig. 21C shows the growth curves from both plates, the growth model dynamics inferred on the first plate alone, and the growth curve produced by the final chosen parameters. Note that our value of R_0 is compatible with the final density of cells reported in previous range expansion studies in similar swim assays (*36*, *39*).

We repeat the same procedure for *E. coli* mutants W1485(F9) and W1485(F10), estimating their parameters in the 0.3% agar plate where they first were sampled, see Supplementary Fig. 22A where the double-front *E. coli* W1485(F10) strain expands on the left of the lysis zone while the single-front *E. coli* W1485(F9) strain drives the dynamics on the right. Extracting the radial intensity profiles along the yellow segments (Supplementary Fig. 22) and then repeating the Monod growth parameters inference yields $r = 1.6 \pm 0.3$ hr^{-1}, $K = 5 \pm 0.08 \cdot 10^9$ CFU/mL and $R_0 = 2.5 \pm 0.03 \cdot 10^9$ CFU/mL for *E. coli* W1485(F9) and $r = 1.2 \pm 0.4$ hr^{-1}, $K = 5 \pm 0.08 \cdot 10^9$ CFU/mL and $R_0 = 2.3 \pm 0.04 \cdot 10^9$ CFU/mL for *E. coli* W1485(F10) (Supplementary Fig. 22C). As final parameters of the full model in Box 1 we choose both for *E. coli* W1485(F9) and *E. coli* W1485(F10) $r = 1.6 \pm 0.3$ hr^{-1} and $K = 5 \pm 0.08 \cdot 10^9$ CFU/mL. In principle these two mutants can have different life traits, but we decided to use common parameters within a standard deviation of the inferred value to avoid overfitting. For the same reason, given variability in the swim assay preparation, we select a common $R_0 = 3.5 \cdot 10^9$ CFU/mL for all three bacteria strains.

Note that for brevity in the equations we rescaled resource densities in unit of CFU/mL, since scalar transformations between units do not impact the model dynamics provided that all parameters are rescaled accordingly. In some cases it can be useful to express all chemical concentrations, including resources, in μg/mL. This can be done based on a conversion factor indicating the amount of μg of resources constituting a cell, the yield ϵ. *E. coli* cells typically contain ~ 200 fg of carbon (*6*), therefore, if half of nutrient weight is due to carbon, it takes about 400 fg of processed nutrients to make up a cell. Assuming a cell metabolizes to growth slightly less than half of nutrients that are taken up, we estimate $\epsilon \approx 10^{-6}\mu$g/cell. In these units, dynamics are unchanged if the Monod constant, the initial resource concentration and the resource consumption rate (now different than bacteria growth rate) are multiplied by ϵ.

We estimate the initial density of bacteria and phage inoculated from the stocks to be around 10^9 per mL (see stocks section in Methods).

- Bacteria motility and sensing

We set the bacteria diffusion constant to $D_B = 1.8 \cdot 10^5 \mu\text{m}^2$/hrs (*36*). Once D_B is fixed, and with the maximum growth rate inferred as above, the sensing responsiveness parameter χ is inferred to match the range expansion speed, that at steady state scales as $v \propto \chi\sqrt{\frac{g(R_0)}{D_B}}$, as detailed in (*36*). To reproduce the 5 mm/hr speed measured in 0.25% agar experiments we set $\chi = 6 \cdot 10^6 \mu\text{m}^2$/hrs. When extrapolating to other agar concentrations we change consistently both D_B and χ. To match the experiments at 0.2% agar in in Fig. 5A we obtain a speed of 8.3 mm/hr with $D_B = 3.2 \cdot 10^5$ and $\chi = 15.2 \cdot 10^6$, while $D_B = 10^5$ and $\chi = 2.9 \cdot 10^6$ yield a speed of 3.5 mm/h matching the experiments at 0.3% agar in Fig. 5B.

The low chemoattractant sensing constant was inferred in (*54*, *74*) to be 3.5μM for the chemoattractant molecule aspartate, (a_1 in our model), which has ≈ 100g/mol $= 0.1\mu$g/(μM$\cdot$mL), yielding approximately $a_- \approx 0.3\mu$g/mL. In the following we will use the same conversion factor from μM to μg/mL for serine, the other main chemoattractant in our swimming assays (*39*) (a_2 in our model), which has a similar molecular mass to aspartate. This conversion leads to a serine Monod constant of approximately $K_2 \approx 5 - 10\,\mu$g/mL (*39*, *72*). The uptake rate for serine $\mu_{2,\max}$ is 3.35 mmol/(g dry weight x hour) during cells exponential growth (*73*). On average, a cell's dry mass amounts to $4 \cdot 10^{-13}$g (*81*), leading to a serine uptake of $3.4 \times 4 \cdot 10^{-13}$ mmol/(cell x hour), or $\mu_{2,\max} = 14 \cdot 10^{-8}\mu$g/(cell x hour). Applying the same conversion, we find an aspartate uptake rate per cell of $\mu_{1,\max} = 2 \cdot 10^{-8}\mu$g/(cell x hour) (*73*). Finally, we estimate two additional life traits for the *E. coli* W1485(F8): the high sensing constant a_+ and the Aspartate Monod constant K_1, which we fit to reproduce the measured radial density profiles. We start from previously inferred $a_+ = 3\mu$g/mL (*36*) and $K_1 = 0.5\mu$g/mL (*39*), and we vary these values until the radial profile of a range expansion simulation resembles the data from the two reference plates used to fit the growth parameters (see Supplementary Fig. 21D). The final value for $a_+ = 10\mu$g/mL is on the same order of magnitude as the previously inferred parameter (*36*), and $K_1 = 3.2\mu$g/mL confirms a lower Monod constant for aspartate than for serine (*39*).

We repeat this procedure to fit chemosensing and motility parameters for *E. coli* mutants W1485(F9) and W1485(F10). In order to reproduce the experimental radial density profiles we set $a_+ = 100\mu$g/mL, which coincidentally corresponds to the value proposed in (*39*) if we apply the conversion ≈ 100g/mol introduced

above, and $K_1 = 1.5\mu$g/mL. To reproduce the new mutants' profiles we also needed to adjust the serine Monod constant K_2 from 10 to 3μg/mL and the aspartate uptake rate $\mu_{1,\max}$ from $2 \cdot 10^{-8}$ to $3 \cdot 10^{-8}\mu$g/(cell x hour). This new set of chemosensing parameters yields the bacteria density profiles in Supplementary Fig. 22D, which closely reproduce the data. These radial curves are produced by simulations with $D_B = 10^5\mu\text{m}^2$/hrs and $\chi = 2.5 \cdot 10^6 \mu\text{m}^2$/hrs, as these values, similar to those used for *E. coli* W1485(F8) in Fig. 5B, yield the $\sim$ 4mm/hr speed measured in the 0.3% agar plate in Supplementary Fig. 22A. To recover the $\sim$ 6.5mm/hr speed observed in 0.25% agar experiments for strain *E. coli* W1485(F10)(Fig. 5C) and *E. coli* W1485(F9) (Fig. 3B) we revert to $D_B = 1.8 \cdot 10^5\mu\text{m}^2$/hrs (*36*) and $\chi = 4 \cdot 10^6\mu\text{m}^2$/hrs – corresponding to the values reported in Table S1. Finally, to obtain the fast 7.3 mm/hr range expansion speed measured in Fig. 3A we set $D_B = 2.7 \cdot 10^5\mu\text{m}^2$/hrs and $\chi = 5.5 \cdot 10^6\mu\text{m}^2$/hrs. Other than the motility parameters to match the range expansion speeds measured in individual plates, given variations in the swim assay preparation, we assume for simplicity that strains *E. coli* W1485(F9) and *E. coli* W1485(F10) share all parameters, with *E. coli* W1485(F9) only responding to serine gradients (equivalent to $a_1 = 0$).

- Viral infection

  We select plausible values that are commonly used in phage-bacteria ecology studies to fix a few phage parameters, namely the viral decay ω from (*75*), phage diffusion constant $D_P = 10^4$ μm^2/hrs from (*76*), and the number of infection steps leading to cell lysis $L = 10$ from (*39*, *41*), which leads to a phage infection time variability in line with empirical estimates (*44*). We vary the values of phage adsorption rate ϕ, lysis rate η and burst size β, selecting the values yielding the closest reproduction of the experimental dynamics, reported in Table S1, which qualitatively agree with previously reported phage infection parameters for T7 and T4 (*75*).

S3. FITTED PARAMETER VALUES: COMPARISON WITH LITERATURE AND ROBUSTNESS ANALYSIS

Among the parameters estimated above, a subset does not find a quantitative match in literature in comparable experimental conditions – in these cases, a reference is not present in the "Source" column of Table S1. Here, we compare these parameters with previously published ones for transparency, while noting differences in experimental conditions. As mentioned at the beginning of Section S2, many of our parameters represent phenomenological terms driving population dynamics rather than mechanistic ground-truths. In this context, the quantitative agreement between our experiments and model justifies differences compared to previously published parameter values. To quantify the impact of such differences on the model dynamics we performed several robustness analyses, summarized here, varying fitted parameters. We organize the following using the same bullet points denoting different processes as in the previous parameters estimation Section.

- Bacteria growth

  The maximum bacteria growth rate r was directly measured in nutrient-rich media between 1.1 and 1.4 hrs^{-1} (and below 1.1 hrs^{-1} in minimal media) (*36*), and was indirectly inferred as 2.8 hrs^{-1} in (*39*) in soft agar experiments with LB medium. Our fitted r values sit in between this range. Additionally, Supplementary Figure 14 C shows that T7 PhASER dynamics are robust to a 4-fold increase in growth rate, comfortably bracketing the value inferred in (*39*) (more details on the effects of modifying nutrients quality and assumptions are given in Section S4). The Monod constant K was inferred in spatial agar assays with LB as about 10^9 CFU/mL (*39*) (upon converting to our units). While our primary values are 5-7 times higher, simulations lowering K to the same value of 10^9 CFU/mL produce PhASERs both for T7 and T4 (Supplementary Figure 11 C and D respectively) with minor quantitative differences compared to our data.

- Bacteria motility and sensing

  The fitted aspartate Monod constants K_1, necessary to quantitatively reproduce our measured radial bacteria density profiles in Supplementary Figures 21 and 22, is on the same order of magnitude as inferred in LB soft agar assays (*39*). As described above, once we fix all other bacteria parameters, the sensing responsiveness parameter χ is inferred to reproduce our measured range expansion speed. The values we inferred from experiments across conditions and strains range from $2.5 \cdot 10^6$ to $15.2 \cdot 10^6\mu\text{m}^2$/hrs, producing the consistent formation of PhASERs at front speeds spanning 3.5 to 8.3 mm/hrs (see Figure 5A,B). The lower end of this range is compatible with the $\chi \sim 1.5 \cdot 10^6\mu\text{m}^2$/hrs inferred in (*36*, *39*), as are the front speeds measured in our experiments.

- Viral infection

  Moving to phage infection parameters, we note that typically phage life-history traits are quantified in liquid cultures. Therefore we cannot make direct comparisons for T7 and T4 phage parameters in the same environment as our experiments, where a number of factors ranging from spatial structure to nutrient dynamics could impact

quantitative phage traits. With this caveat in mind, phage life-history traits were reported in (*75*) as $\phi = 3 \cdot 10^{-8}$ mL/(hrs $\cdot$ PFU), $\eta = 2.6$ hrs^{-1}, $\beta = 150$ PFU/CFU for phage T4, and $\phi = 1.8 \cdot 10^{-7}$ mL/(hrs $\cdot$ PFU), $\eta = 4.6$ hrs^{-1}, $\beta = 260$ PFU/CFU for phage T7. As mentioned above, different spatial settings could produce significant differences in the inferred ϕ value which implicitly accounts for diffusion/motility processes affecting phage-bacteria encounters, as ϕ scales linearly with phage diffusion constant (*35*). Phage adsorption rate is also known to significantly vary with cell surface in different growth conditions (*82*). More broadly, phage life-history traits vary widely with nutrient availability and hosts growth rates (*82*, *83*), a theme investigated in detail in Section S4. Modulating bacteria growth rates between 0.5 and 1.2 hrs^{-1} approximate burst sizes were reported between 40 and 120 PFU/CFU for phage T7 (estimated from Fig. 2 of (*83*)). For phage T4, similar bacteria growth rates yield burst sizes between 20 and 60 PFU/CFU, and up to 110 PFU/CFU with a growth rate approaching 1.8 hrs^{-1} (*82*).

Motivated by these large variations in measured phage life-history traits, we performed a robustness analysis varying phage infection parameters. First, we explored the simulated dynamics of phage transported by *E. coli* W1485(F9) when varying the magnitude of a linear adsorption rate and of lysis rate, spanning two orders of magnitude around the parameters inferred for phage T7. Supplementary Fig. 7 shows that for extremely low adsorption rates and lysis rates (corresponding to an average infection time of 1 hour) the phage infections are unable to sustain PhASERs. Increasing either adsorption or lysis rates (up to 4 minutes average infection period) produce stable PhASERs with a wider and wider lysis region, until the chemotactic fronts at its sides do not reconverge within our experiment's scales. Yet, within these two extreme limits, PhASER emergence shows a remarkable robustness to 10-fold variations in viral life traits, up to ϕ values close to those reported in (*75*). Then we repeated the same procedure using the saturating adsorption profile of phage T4, spanning one order of magnitude around the corresponding η and ϕ parameters (see Supplementary Figure 8). The realization of the observed experimental dynamics with residual cells inside narrow PhASERs appears to be more sensitive on these parameters values. High adsorption and lysis rates offset the nonlinear phage saturation profile and produce PhASERs that present T7-like features – i.e. initial curved fronts divergence and vanishing cell densities inside PhASERs. Finally, in Supplementary Figure 9 we increased the burst size β both for phage T7 (panel A) and phage T4 (panel B), bracketing previously measured values (*75*, *82*, *83*). With $\beta = 150$ PFU/CFU we recover qualitatively similar PhASER dynamics. A higher $\beta = 250$ PFU/CFU yields T7 lysis regions that do not reconverge within our experiment's scales, while T4 dynamics start presenting quantitative PhASER features typically associated with phage T7 as already noted for Supplementary Figure 8.

S4. IMPACT OF CORE MODEL INGREDIENTS

Here we discuss in detail the remaining functional components constituting our mathematical model that were not addressed in Sections S2 and S3. To clarify the processes enabling the emergence of PhASERs, we tested the model behavior when modifying its core ingredients.

- **Phage discreteness threshold**
  The model relies on infection-mediated feedback determining the dependence of infection properties on resources and phage densities. We modulate all phage reaction terms by a nonlinear function $T(P) = \frac{P^M}{P^M + \Theta^M}$ with $M = 10$ to ensure that our continuous PDE model does not integrate early phage proliferation from nonphysical low phage densities. According to this 'single-phage form factor' phage rates sharply go to 0 when phage densities are lower than 1 virion per lattice site $P < \Theta = \frac{1}{V_l}$, with $V_l = 0.5 \cdot 10^{-4}$ being the volume of a site (see Section S1). Considering a simple well-mixed phage predation model with phage being washed out at rate α due to decay and spatial transport $\frac{\partial P}{\partial t} = (\beta - 1)\phi S P T(P) - \alpha P$, phage invading a phage-free system with S^* bacteria grow ($\frac{\partial P}{\partial t} > 0$) if
  $$P > \frac{\Theta}{\left(\frac{\phi(\beta-1)S^*}{\alpha} - 1\right)^{\frac{1}{M}}}.$$
  Therefore, with $M \gg 1$, phage can invade a dense bacteria lawn only above densities $P \gtrsim \Theta$, while with $T(P) = 1$ even a fraction of a virion would result in phage proliferation.
- **Non-exponential latent period distribution**
  Another key ingredient in our model is that infected cells progress through $L = 10$ infection stages between phage adsorption and lysis, corresponding to an Erlang distribution of phage infection times with a Coefficient of Variation (CV) of $1/\sqrt{10} \sim 0.3$ (*41*, *84*). To address the necessity of this ingredient we simulated the model

dynamics with $L = 1$ (*38*), which corresponds to an exponentially distributed latent period, therefore increasing the CV to 1 while keeping the mean of the latent period distribution fixed. We found that an exponentially distributed infection time does not reproduce the experimental dynamics, as the high probability of lysis after a vanishing infection time produces a sideways expansion of the T7 lysis zone that outpaces the chemotactic range expansion of adjacent uninfected bacteria. See Supplementary Fig. 4A showing a simulation where *E. coli* W1485(F8) confronts phage T7, with the same parameters as in Fig. 4 except for the latent period variance.

To characterize further the impact of lysis time distributions, we tested the effect of an increasing number of infected stages on $24cm$ plates dynamics simulated with the parameters otherwise inferred for either phage T7 or T4 (Supplementary Figure 5). Again, assuming an exponentially distributed latent period leads to a lysis zone that keeps expanding outwards for more than 30 cm, diverging drastically from our empirical observations, as shown in Supplementary Figure 5, panel A for T7 and D for T4. Increasing the number of infected stages produces a narrower unimodal latent period distribution, with a smaller Coefficient of Variation (decreasing as $1/\sqrt{L}$) around the fixed average lysis time. This yields a faster reconvergence of the initial lysis zone, although with $L = 2$ we observe an instability of T7 PhASERs to fronts merging (panel B). This outcome is impacted by phage life history traits, since T4 parameters with $L = 2$ produce a stable PhASER (panel E). Introducing 3 infected stages was sufficient to produce a long-range PhASER regardless of phage parameters (panel C for T7 and F for T4), therefore the dynamics stabilize well ahead of the $L = 10$ value employed in the main text. This finding is consistent with empirical measurements of the latent period's CV, which are well below 1 and are compatible our choice of 10 infected stages (*44*). Note that smaller $9cm$-diameter plates (dotted blue circle in Supplementary Figure 5) do not permit sufficient space to assess the curvature of initial lysis boundaries (*38*) making it hard to distinguish between exponential and peaked distributions, and consequently prevent the characterization of PhASERs stability.

- **Coupling between resources and phage infection**

In our model the transition rate from phage adsorption to lysis is coupled to cell metabolism through resource concentration as $\eta(R) = \eta\frac{R}{R+K}$ as is the burst size $\beta(R) = 1 + \beta\frac{R}{R+K}$, which are therefore linearly related to bacteria growth rate. Supplementary Fig. 4B shows a simulation with *E. coli* W1485(F8) *vs* T7 where phage infections do not slow down as resources are depleted, with $\eta(R) = \eta/3$ ensuring the same basal lysis rate as in Fig. 4 of the main text since $K = 2R_0$. After phage infections are transported by the range expansion front (gray overlay showing 2h-spaced fronts), phage slowly consume the bacterial lawn through a constant expansion of the lysis region behind the front.

Then, we sought to test the qualitative impact of introducing a nonlinear dependence of lysis rate and burst size on bacteria growth rate. A quantitative characterization of this dependence in spatial environments where bacteria dynamically run out of nutrients is currently not available. Therefore we turned our attention to measurements of the dependence of phage life traits with hosts growth rates in well-mixed cultures where bacteria grow at steady state. While noting the different conditions with respect to our experiments, which could impact the quantitative growth-dependent phage traits functions, this choice allows us to qualitatively test nonlinear functions informed by empirical observations. Specifically, we leverage the work presented in (*47*), where the authors inferred the dependence of phage life traits with host growth rate from previous experiments on phage T7 (*83*) and T4 (*82*, *85*). An empirically-informed evolutionary steady state analysis in (*47*) yields the latent period (reported in minutes therein) as a function of host growth rate $\tilde{r}$ normalized by the maximum growth rate measured for each phage r, as:

$$l(r_{\text{norm}}) = \frac{1}{w} + E_\infty + E_0\, e^{-\alpha_E r_{\text{norm}}},$$

and a burst size given by:

$$\beta(r_{\text{norm}}) = \frac{M_\infty}{w} \cdot \frac{1}{1 + e^{-\alpha_M (r_{\text{norm}} - M_0)}},$$

with $r_{\text{norm}} = \frac{\tilde{r}}{r}$. In our model, $r_{\text{norm}} = \frac{R}{R+K}$, which decreases as resources R are consumed. The parameters in these function were inferred in (*47*) from (*83*) for phage T7 as $E_\infty = 15.33$ min, $E_0 = 82.71$ min, $\alpha_E = 5.9$, $M_\infty = 6.7$ PFU/(CFU $\cdot$ min), $M_0 = 0.64$, $\alpha_M = 9.7$, and from (*82*, *85*) for phage T4 as $E_\infty = 18.26$ min, $E_0 = 94.18$ min, $\alpha_E = 7.76$, $M_\infty = 47.32$ PFU/(CFU $\cdot$ min), $M_0 = 0.65$, $\alpha_M = 12.03$. These functions depend on a free parameter w , which we set so that the initial lysis rate, with resources at the initial concentration R_0 (Table S1), is the same as in the main text simulations. In other words, we set w so that $l\left(r_{\text{norm}} = \frac{R_0}{R_0+K}\right)/60 = \left(\eta\frac{R_0}{R_0+K}\right)^{-1}$, where the term 60 rescales the time units from minutes to hours, consistently with our units. This

choice allows us to test the impact of adding this nonlinearity, without affecting the range of the lysis rates realized in our simulations based on the resource concentration dynamics. This leads to a nonlinear growth-dependent lysis rate:

$$\eta(r_{\mathrm{norm}}) = \frac{\eta \frac{R_0}{R_0+K}}{1 + \frac{\eta}{60}\frac{R_0}{R_0+K} E_0 \left(e^{-\alpha_E r_{\mathrm{norm}}} - e^{-\alpha_E \frac{R_0}{R_0+K}} \right)}, \tag{S1}$$

where the constant η in the right hand side denotes the parameter specified in Table S1. Similarly, to consistently renormalize the burst size to the correct initial value, we set w so that $\beta\left(r_{\mathrm{norm}} = \frac{R_0}{R_0+K}\right) = 1 + \beta\frac{R_0}{R_0+K}$. This yields a nonlinear burst size:

$$\beta(r_{\mathrm{norm}}) = \left(1 + \beta\frac{R_0}{R_0+K}\right) \frac{1 + e^{-\alpha_M\left(\frac{R_0}{R_0+K} - M_0\right)}}{1 + e^{-\alpha_M (r_{\mathrm{norm}} - M_0)}}, \tag{S2}$$

where the right hand side constant β is specified in Table S1.

Supplementary Figure 10 shows the results of spatial simulations employing these nonlinear phage traits functions. Panels A and B respectively show eq. (S1) and eq. (S2) (full lines), compared to the linear function employed in the main text (dashed lines), both for phage T7 (red) and T4 (blue). The x-axis spans the normalized growth rate range realized in the simulations based on the resources dynamics parameters – *i.e.* $[0, \frac{R_0}{R_0+K}]$. Despite the quantitative functional differences, these nonlinear phage traits produce PhASER dynamics very similar to the linear traits presented in Figure 3 in the main text (panel C for phage T7 and panel D for phage T4).

To test the impact of nonlinear lysis rate and burst size even further, we decrease the Monod constant K from $5 \cdot 10^9$ CFU/mL to 10^9 CFU/mL, almost doubling $\frac{R_0}{R_0+K}$ and consequently the range of normalized growth rates and phage traits spanned in the spatial simulations. This choice exacerbates the nonlinearities in lysis rate and burst size in equations (S1) and (S2) (see Supplementary Figure 11 A and B respectively). First, we run simulations with this modified parameter using the original linear dependence (dashed lines in Supplementary Figure 11 A and B) of lysis rate and burst size with growth rate. The spatial simulations results are shown in Supplementary Figure 11 C for phage T7 and in Supplementary Figure 11 D for phage T4. As already mentioned in the previous Section, the 5-fold reduction in K produces the typical qualitative PhASER dynamics as in the main text both for phage T7 and T4. Then, we run new simulations using the nonlinear dependence expressed in equations (S1) and (S2) (full lines in Supplementary Figure 11 A and B), both for phage T7 (Supplementary Figure 11 E) and T4 (Supplementary Figure 11 F). The nonlinearities in lysis rate and burst size, more pronounced in these conditions compared to those simulated in Supplementary Figure 10, still do not affect the emergence of stable PhASERs, despite small quantitative differences in the simulated dynamics.

- **Nonlinear T4 phage adsorption rate and phage infection strength**

  The simulations reproducing phage T4 experimental large-scale dynamics presented in the main text employ an adsorption profile saturation at high phage densities $F(P) = \phi\frac{P}{1+P/P_c}$ with $P_C = 10^7$ PFU/mL (*84*, *86*), following Michaelis-Menten reaction kinetics (*45*) which mediate inter-phage competition processes such as lysis inhibition (*46*). This saturation profile helps capture more features of T4 PhASERs, such as the presence of a low density of cells coexisting with phage within the lysis region. Yet, we highlight here that we can reproduce most of the qualitative features of T4 PhASERs with a simpler linear adsorption $F(P) = \phi P$ similar to simulated T7 dynamics. Indeed, Supplementary Fig. 17 shows T4-like simulated dynamics resulting from a linear adsorption profile with $\phi = 2 \cdot 10^{-9}$ mL/(hrs · PFU), $\eta = 5$ hrs^{-1} and $\beta = 50$ PFU/cell, while the rest of the parameters are fixed to the *E. coli* W1485(F8) bacterium strain values reported in Table S1. These ingredients yield a narrow T4-like PhASER propagated over long distances (Supplementary Fig. 17) which transfers to coalescing colonies expanding at different speeds provided that the angle of incidence between the merging fronts is not too big (Supplementary Fig. 17), as in the infection transport dynamics presented in Fig. 6 in the main text.

- **Chemically distinct nutrients and attractants**

  We tested the model predictions merging nutrients and attractants into the same state variable (*38*), ignoring the chemical complexity of the LB medium. To do so, we rescaled the parameters setting the concentration band of maximum sensitivity, a_- and a_+, multiplying them by the ratio of carrying capacities K/K_2 to match the order of magnitude of resource dynamics, and we set $\chi = 5 \cdot 10^7 \mu\text{m}^2$/hrs to keep the range expansion speed comparable to experiments. Supplementary Figure 6 shows that conflating nutrients and attractants leads to qualitatively different phage propagation dynamics. Keeping the rest of the parameters unchanged with respect to those inferred for T7, including the average later period ($\eta = 5$/hr) (panels A,B,C), we see that bacteria wash away all phage and invade the whole plate, due to the fact that the bacteria population saturates to carrying

capacity and limits lysis right behind the front (see (*36*)). At intermediate time snapshots before bacteria fill the infected sector (A,B) the boundaries of the phage-bacteria interface appear to be straight as reported by (*38*). This outcome is even clearer when we increase the lysis rate η just enough to stop bacteria invasion at the center of the plate, setting $\eta = 10/\text{hr}$ (D,E,F), which leads to straight boundaries throughout the simulated dynamics.

- **Biomass conversion and nutrients quality**

  To complement our experimental investigation on the impact of environmental variations (Figure 5), we extended the numerical exploration of the model addressing its robustness to nutrients quality, another environment feature, focusing on *E. coli* strain W1485(F9). To do so, we vary the biomass conversion factor ϵ (yield) between nutrients measured in μg/mL and cells measured in CFU/mL, introduced in Section S2. As noted when we discussed our parametrization of bacterial growth, choosing a different unit of measure for nutrients, expressed in CFU/mL in the main text, does not affect the model dynamics.

  Setting $\epsilon = 5 \cdot 10^{-7} \mu\text{g/cell}$ halves the yield with respect to the original parameters (lower nutrient quality or metabolic efficiency, corresponding to a slower initial doubling time of 2.6 hours) and the T7 phage are transported with a widened initial lysis region (Supplementary Figure 14 A). Supplementary Figure 14 B reproduces the main T7 simulations with $\epsilon = 10^{-6} \mu\text{g/cell}$ for comparison. When the yield is set to 4 times the original parameter cells grow faster, corresponding to a 20 minutes initial doubling time, and a PhASER emerges with a narrower lysis region (Supplementary Figure 14 C). Note that as predicted by (*36*), changing the growth rate affects the chemotactic range expansion speed. The high yield conditions in panel C produce a fast 15 mm/hr range expansion, which is comparable to chemotactic front speeds reported in liquid media (*54*). This result suggests that the model qualitative dynamics are robust to nutrient quality perturbations.

Supplementary Movie 1. Timelapse sequence of an expanding *E. coli* W1485(F8) population and phage T7 PhASER, corresponding to the snapshots shown in Figure 1A.

Supplementary Movie 2. Timelapse sequence of an expanding *E. coli* W1485(F9) population and phage T7 PhASER, corresponding to the snapshots shown in Figure 1C and Figure 3A left.

Supplementary Movie 3. Timelapse sequence of a model simulation with parameters inferred for *E. coli* W1485(F9) and phage T7 (see Supplementary Text), corresponding to the snapshot shown in Figure 3A right.

Supplementary Movie 4. Timelapse sequence of an expanding *E. coli* W1485(F9) population and phage T4 PhASER, corresponding to the snapshot shown in Figure 3B left.

Supplementary Movie 5. Timelapse sequence of a model simulation with parameters inferred for *E. coli* W1485(F9) and phage T4 (see Supplementary Text), corresponding to the snapshot shown in Figure 3B right.

Supplementary Movie 6. Timelapse sequence of a model simulation with parameters inferred for *E. coli* W1485(F8) and phage T4 (see Supplementary Text), where two bacteria colonies expand carrying phage T4 so that PhASERs intersect at a 60 degrees angle. This movie shows detailed information on the simulated dynamics for the density of total (grey) and infected (blue) cells and the density of phage (red). The two green lines represent attractant density isolines at half the initial value and at the lower sensing threshold. The thin yellow line, close to the front, denotes the isoline where the chemosensing response is at 80% its maximum. Quivers represent the chemotactic flux along this line, colored according to the fraction of infected cells. This movie corresponds to the snapshots shown in Figure 6A right and Figure 6B.

Supplementary Movie 7. Timelapse sequence of a model simulation with parameters inferred for *E. coli* W1485(F8) and phage T4 (see Supplementary Text), where two bacteria colonies expand carrying phage T4 so that PhASERs intersect at a 120 degrees angle. This movie shows detailed information on the simulated dynamics for the density of total (grey) and infected (blue) cells and the density of phage (red). The two green lines represent attractant density isolines at half the initial value and at the lower sensing threshold. The thin yellow line, close to the front, denotes the isoline where the chemosensing response is at 80% its maximum. Quivers represent the chemotactic flux along this line, colored according to the fraction of infected cells. This movie corresponds to the snapshots shown in Figure 6C right and Figure 6D.

Supplementary Movie 8. Timelapse sequence of an experiment where a T4 PhASER on *E. coli* W1485(F8) collides with the migrating front of a much faster *E. coli* strain W1485(F10). This movie corresponds to the snapshot shown in Figure 6E top.

Supplementary Movie 9. Timelapse sequence of a model simulation where a T4 PhASER on *E. coli* W1485(F8) collides with the migrating front of a much faster *E. coli* strain W1485(F10). The model parameters generating this movie, inferred for each featured strain, are reported in Supplementary Text Table S1. This movie corresponds to the snapshot shown in Figure 6E bottom.

Supplementary Movie 10. Timelapse sequence of an expanding *E. coli* W1485(F9) population + phage T7 PhASER, showing example processed image sequences featuring raw snapshots (top left), blue channel (bottom left), differential (top right), and extracted fronts (bottom right).

REFERENCES

1. S. T. Abedon, *Bacteriophage Ecology: Population Growth, Evolution and Impact of Bacterial Viruses* (Cambridge Univ. Press, 2008).

2. M. Delbruck, The growth of bacteriophage and lysis of the host. *J. Gen. Physiol.* **23**, 643–660 (1940).

3. H. B. Judson, *The Eighth Day of Creation* (Simon & Schuster, 1979).

4. E. L. Ellis, M. Delbruck, The growth of bacteriophage. *J. Gen. Physiol.* **22**, 365–384 (1939).

5. B. R. Levin, F. M. Stewart, L. Chao, Resource-limited growth, competition, and predation: A model and experimental studies with bacteria and bacteriophage. *Am. Nat.* **111**, 3–24 (1977).

6. J. S. Weitz, *Quantitative Viral Ecology: Dynamics of Viruses and Their Microbial Hosts*, Monographs in Population Biology (Princeton Univ. Press, 2016).

7. C. H. Wigington, D. L. Sonderegger, C. P. Brussaard, A. Buchan, J. F. Finke, J. Fuhrman, J. T. Lennon, M. Middelboe, C. A. Suttle, C. Stock, W. H. Wilson, K. E. Wommack, S. W. Wilhelm, J. S. Weitz, Re-examination of the relationship between marine virus and microbial cell abundances. *Nat. Microbiol.* **1**, 15024 (2016).

8. F. Rohwer, R. V. Thurber, Viruses manipulate the marine environment. *Nature* **459**, 207–212 (2009).

9. S. Roux, J. R. Brum, B. E. Dutilh, S. Sunagawa, M. B. Duhaime, A. Loy, B. T. Poulos, N. Solonenko, E. Lara, J. Poulain, S. Pesant, S. Kandels-Lewis, C. Dimier, M. Picheral, S. Searson, C. Cruaud, A. Alberti, C. M. Duarte, J. M. Gasol, D. Vaqué, P. Bork, S. G. Acinas, P. Wincker, M. B. Sullivan, Ecogenomics and potential biogeochemical impacts of globally abundant ocean viruses. *Nature* **537**, 689–693 (2016).

10. R. A. Edwards, A. A. Vega, H. M. Norman, M. Ohaeri, K. Levi, E. A. Dinsdale, O. Cinek, R. K. Aziz, K. McNair, J. J. Barr, K. Bibby, S. J. J. Brouns, A. Cazares, P. A. De Jonge, C. Desnues, S. L. D. Muñoz, P. C. Fineran, A. Kurilshikov, R. Lavigne, K. Mazankova, D. T.

McCarthy, F. L. Nobrega, A. R. Muñoz, G. Tapia, N. Trefault, A. V. Tyakht, P. Vinuesa, J. Wagemans, A. Zhernakova, F. M. Aarestrup, G. Ahmadov, A. Alassaf, J. Anton, A. Asangba, E. K. Billings, V. A. Cantu, J. M. Carlton, D. Cazares, G.-S. Cho, T. Condeff, P. Cortés, M. Cranfield, D. A. Cuevas, R. De La Iglesia, P. Decewicz, M. P. Doane, N. J. Dominy, L. Dziewit, B. M. Elwasila, A. M. Eren, C. Franz, J. Fu, C. Garcia-Aljaro, E. Ghedin, K. M. Gulino, J. M. Haggerty, S. R. Head, R. S. Hendriksen, C. Hill, H. Hyöty, E. N. Ilina, M. T. Irwin, T. C. Jeffries, J. Jofre, R. E. Junge, S. T. Kelley, M. K. Mirzaei, M. Kowalewski, D. Kumaresan, S. R. Leigh, D. Lipson, E. S. Lisitsyna, M. Llagostera, J. M. Maritz, L. C. Marr, A. McCann, S. Molshanski-Mor, S. Monteiro, B. Moreira-Grez, M. Morris, L. Mugisha, M. Muniesa, H. Neve, N. Nguyen, O. D. Nigro, A. S. Nilsson, T. O'Connell, R. Odeh, A. Oliver, M. Piuri, A. J. P. Ii, U. Qimron, Z.-X. Quan, P. Rainetova, A. Ramírez-Rojas, R. Raya, K. Reasor, G. A. O. Rice, A. Rossi, R. Santos, J. Shimashita, E. N. Stachler, L. C. Stene, R. Strain, R. Stumpf, P. J. Torres, A. Twaddle, M. U. Ibekwe, N. Villagra, S. Wandro, B. White, A. Whiteley, K. L. Whiteson, C. Wijmenga, M. M. Zambrano, H. Zschach, B. E. Dutilh, Global phylogeography and ancient evolution of the widespread human gut virus crAssphage. *Nat. Microbiol.* **4**, 1727–1736 (2019).

11. P. Manrique, B. Bolduc, S. T. Walk, J. Van Der Oost, W. M. De Vos, M. J. Young, Healthy human gut phageome. *Proc. Natl. Acad. Sci. U.S.A.* **113**, 10400–10405 (2016).

12. A. Chevallereau, B. J. Pons, S. van Houte, E. R. Westra, Interactions between bacterial and phage communities in natural environments. *Nat. Rev. Microbiol.* **20**, 49–62 (2022).

13. B. J. M. Bohannan, R. E. Lenski, Effect of resource enrichment on a chemostat community of bacteria and bacteriophage. *Ecology* **78**, 2303–2315 (1997).

14. B. J. M. Bohannan, R. E. Lenski, Linking genetic change to community evolution: Insights from studies of bacteria and bacteriophage. *Ecol. Lett.* **3**, 362–377 (2000).

15. L. Chao, B. R. Levin, F. M. Stewart, A complex community in a simple habitat: An experimental study with bacteria and phage. *Ecology* **58**, 369–378 (1977).

16. S. E. Luria, M. Delbrück, Mutations of bacteria from virus sensitivity to virus resistance. *Genetics* **28**, 491–511 (1943).

17. R. E. Lenski, Coevolution of bacteria and phage: Are there endless cycles of bacterial defenses and phage counterdefenses? *J. Theor. Biol.* **108**, 319–325 (1984).

18. C. O. Flores, J. R. Meyer, S. Valverde, L. Farr, J. S. Weitz, Statistical structure of host–phage interactions. *Proc. Natl. Acad. Sci. U.S.A.* **108**, E288–E297 (2011).

19. J. R. Meyer, D. T. Dobias, J. S. Weitz, J. E. Barrick, R. T. Quick, R. E. Lenski, Repeatability and contingency in the evolution of a key innovation in phage lambda. *Science* **335**, 428–432 (2012).

20. J. M. Borin, J. J. Lee, A. Lucia-Sanz, K. R. Gerbino, J. S. Weitz, J. R. Meyer, Rapid bacteria-phage coevolution drives the emergence of multi-scale networks. *Science* **382**, 674–678 (2023).

21. A. Buckling, P. B. Rainey, Antagonistic coevolution between a bacterium and a bacteriophage. *Proc. R. Soc. Lond. Ser. B. Biol. Sci.* **269**, 931–936 (2002).

22. A. Betts, O. Kaltz, M. E. Hochberg, Contrasted coevolutionary dynamics between a bacterial pathogen and its bacteriophages. *Proc. Natl. Acad. Sci. U.S.A.* **111**, 11109–11114 (2014).

23. A. Gupta, S. Peng, C. Y. Leung, J. M. Borin, S. J. Medina, J. S. Weitz, J. R. Meyer, Leapfrog dynamics in phage-bacteria coevolution revealed by joint analysis of cross-infection phenotypes and whole genome sequencing. *Ecol. Lett.* **25**, 876–888 (2022).

24. C. Tropini, K. A. Earle, K. C. Huang, J. L. Sonnenburg, The gut microbiome: Connecting spatial organization to function. *Cell Host Microbe* **21**, 433–442 (2017).

25. B. Kerr, C. Neuhauser, B. J. M. Bohannan, A. M. Dean, Local migration promotes competitive restraint in a host–pathogen “tragedy of the commons”. *Nature* **442**, 75–78 (2006).

26. B. Kerr, M. A. Riley, M. W. Feldman, B. J. M. Bohannan, Local dispersal promotes biodiversity in a real-life game of rock-paper-scissors. *Nature* **418**, 171–174 (2002).

27. R. S. Eriksen, N. Mitarai, K. Sneppen, Sustainability of spatially distributed bacteria-phage systems. *Sci. Rep.* **10**, 3154 (2020).

28. W. Wei, S. M. Krone, Spatial invasion by a mutant pathogen. *J. Theor. Biol.* **236**, 335–348 (2005).

29. J. J. Bull, K. A. Christensen, C. Scott, B. R. Jack, C. J. Crandall, S. M. Krone, Phage-bacterial dynamics with spatial structure: Self organization around phage sinks can promote increased cell densities. *Antibiotics (Basel)* **7**, 8 (2018).

30. B. Ashby, S. Gupta, A. Buckling, Spatial structure mitigates fitness costs in host-parasite coevolution. *Am. Nat.* **183**, E64–E74 (2014).

31. S. Heilmann, K. Sneppen, S. Krishna, Coexistence of phage and bacteria on the boundary of self-organized refuges. *Proc. Natl. Acad. Sci. U.S.A.* **109**, 12828–12833 (2012).

32. E. L. Simmons, M. C. Bond, B. Koskella, K. Drescher, V. Bucci, C. D. Nadell, Biofilm structure promotes coexistence of phage-resistant and phage-susceptible bacteria. *mSystems* **5**, e00877-19 (2020).

33. D. H. Janzen, Herbivores and the Number of Tree Species in Tropical Forests. *American Naturalist* **104**, 501–528 (1970).

34. J. H. Connell, "On the role of natural enemies in preventing competitive exclusion in some marine animals and in rain forest trees," in *Dynamics of Populations*, P. J. D. Boer, G. R. Gradwell, eds. (Pudoc, 1970), pp. 298–312.

35. H. C. Berg, E. M. Purcell, Physics of chemoreception. *Biophys. J.* **20**, 193–219 (1977).

36. J. Cremer, T. Honda, Y. Tang, J. Wong-Ng, M. Vergassola, T. Hwa, Chemotaxis as a navigation strategy to boost range expansion. *Nature* **575**, 658–663 (2019).

37. A. V. Narla, J. Cremer, T. Hwa, A traveling-wave solution for bacterial chemotaxis with growth. *Proc. Natl. Acad. Sci. U.S.A.* **118**, e2105138118 (2021).

38. X. Li, F. Gonzalez, N. Esteves, B. E. Scharf, J. Chen, Formation of phage lysis patterns and implications on co-propagation of phages and motile host bacteria. *PLoS Comput. Biol.* **16**, e1007236 (2020).

39. D. Ping, T. Wang, D. T. Fraebel, S. Maslov, K. Sneppen, S. Kuehn, Hitchhiking, collapse, and contingency in phage infections of migrating bacterial populations. *ISME J.* **14**, 2007–2018 (2020).

40. E. Shaer Tamar, R. Kishony, Multistep diversification in spatiotemporal bacterial-phage coevolution. *Nat. Commun.* **13**, 7971 (2022).

41. N. Mitarai, S. Brown, K. Sneppen, Population dynamics of phage and bacteria in spatially structured habitats using phage λ and *Escherichia coli*. *J. Bacteriol.* **198**, 1783–1793 (2016).

42. J. S. Weitz, J. Dushoff, Alternative stable states in host–phage dynamics. *Theor. Ecol.* **1**, 13–19 (2008).

43. Z. Wang, N. Goldenfeld, Fixed points and limit cycles in the population dynamics of lysogenic viruses and their hosts. *Phys. Rev. E* **82**, 011918 (2010).

44. M. Dominguez-Mirazo, J. D. Harris, D. Demory, J. S. Weitz, Accounting for cellular-level variation in lysis: Implications for virus–host dynamics. *mmBio* **15**, e01376-24 (2024).

45. D. Nabergoj, P. Modic, A. Podgornik, Effect of bacterial growth rate on bacteriophage population growth rate. *Microbiologyopen* **7**, e00558 (2018).

46. T. V. P. Nguyen, Y. Wu, T. Yao, J. T. Trinh, L. Zeng, Y. R. Chemla, I. Golding, Coinfecting phages impede each other's entry into the cell. *Curr. Biol.* **34**, 2841–2853.e18 (2024).

47. M. Choua, J. A. Bonachela, Ecological and evolutionary consequences of viral plasticity. *Am. Nat.* **193**, 346–358 (2019).

48. J. van der Kolk, F. Raßhofer, R. Swiderski, A. Haldar, A. Basu, E. Frey, Anomalous collective dynamics of autochemotactic populations. *Phys. Rev. Lett.* **131**, 088201 (2023).

49. T. B. Jacobson, M. M. Callaghan, D. Amador-Noguez, Hostile takeover: How viruses reprogram prokaryotic metabolism. *Annu. Rev. Microbiol.* **75**, 515–539 (2021).

50. G. Mahmoudabadi, R. Milo, R. Phillips, Energetic cost of building a virus. *Proc. Natl. Acad. Sci. U.S.A.* **114**, E4324–E4333 (2017).

51. E. Djamali, J. D. Nulton, P. J. Turner, F. Rohwer, P. Salamon, Heat output by marine microbial and viral communities. *J. Non-Equilib. Thermodyn.* **37**, 291–313 (2012).

52. J. M. Keegstra, F. Carrara, R. Stocker, The ecological roles of bacterial chemotaxis. *Nat. Rev. Microbiol.* **20**, 491–504 (2022).

53. W. Liu, J. Cremer, D. Li, T. Hwa, C. Liu, An evolutionarily stable strategy to colonize spatially extended habitats. *Nature* **575**, 664–668 (2019).

54. X. Fu, S. Kato, J. Long, H. H. Mattingly, C. He, D. C. Vural, S. W. Zucker, T. Emonet, Spatial self-organization resolves conflicts between individuality and collective migration. *Nat. Commun.* **9**, 2177 (2018).

55. C. M. Taylor, A. Hastings, Allee effects in biological invasions. *Ecol. Lett.* **8**, 895–908 (2005).

56. J. Kayser, C. F. Schreck, M. Gralka, D. Fusco, O. Hallatschek, Collective motion conceals fitness differences in crowded cellular populations. *Nat. Ecol. Evol.* **3**, 125–134 (2019).

57. A. Smith, M. Hunter, S. Bakshi, D. Fusco, Live and let die: Lysis time variability and resource limitation shape lytic bacteriophage fitness. bioRxiv 2025.07.21.665848 [Preprint] (2025); https://doi.org/10.1101/2025.07.21.665848.

58. L. Bergamaschi, N. Mitarai, Stochastic emergence of irregular infection fronts in motile bacteria-phage populations. *Commun. Phys.* **9**, 214 (2026).

59. C. Wedd, T. Yunusov, A. Smith, R. Li, G. Hardo, M. Hunter, R. Majed, D. Fusco, S. Bakshi, Single-cell imaging of the lytic phage life cycle in bacteria. bioRxiv 2024.04.11.588870 [Preprint] (2024); https://doi.org/10.1101/2024.04.11.588870.

60. A. M. Kropinski, A. Mazzocco, T. E. Waddell, E. Lingohr, R. P. Johnson, "Enumeration of bacteriophages by double agar overlay plaque assay," in *Bacteriophages: Methods and Protocols, Volume 1: Isolation, Characterization, and Interactions*, M. R. J. Clokie, A. M. Kropinski, eds. (Humana Press, 2009), pp. 69–76.

61. M. Baym, T. D. Lieberman, E. D. Kelsic, R. Chait, R. Gross, I. Yelin, R. Kishony, Spatiotemporal microbial evolution on antibiotic landscapes. *Science* **353**, 1147–1151 (2016).

62. N. Stroustrup, B. E. Ulmschneider, Z. M. Nash, I. F. López-Moyado, J. Apfeld, W. Fontana, The *Caenorhabditis elegans* lifespan machine. *Nat. Methods* **10**, 665–670 (2013).

63. J. Schindelin, I. Arganda-Carreras, E. Frise, V. Kaynig, M. Longair, T. Pietzsch, S. Preibisch, C. Rueden, S. Saalfeld, B. Schmid, J.-Y. Tinevez, D. J. White, V. Hartenstein, K. Eliceiri, P. Tomancak, A. Cardona, Fiji: An open-source platform for biological-image analysis. *Nat. Methods* **9**, 676–682 (2012).

64. H. C. Berg, *Random Walks in Biology* (Princeton Univ. Press, 1993).

65. A. H. Doermann, Lysis and lysis inhibition with *Escherichia coli* bacteriophage. *J. Bacteriol.* **55**, 257–276 (1948).

66. Y. Tu, T. S. Shimizu, H. C. Berg, Modeling the chemotactic response of *Escherichia coli* to time-varying stimuli. *Proc. Natl. Acad. Sci. U.S.A.* **105**, 14855–14860 (2008).

67. N. Nikolic, V. Anagnostidis, A. Tiwari, R. Chait, F. Gielen, Droplet-based methodology for investigating bacterial population dynamics in response to phage exposure. *Front. Microbiol.* **14**, 1260196 (2023).

68. W. Möbius, A. W. Murray, D. R. Nelson, How obstacles perturb population fronts and alter their genetic structure. *PLoS Comput. Biol.* **11**, e1004615 (2015).

69. M. L. Yap, M. G. Rossmann, Structure and function of bacteriophage T4. *Future Microbiol.* **9**, 1319–1327 (2014).

70. A. Grace, R. Sahu, D. R. Owen, V. A. Dennis, *Pseudomonas aeruginosa* reference strains PAO1 and PA14: A genomic, phenotypic, and therapeutic review. *Front. Microbiol.* **13**, 1023523 (2022).

71. V. Krylov, M. Bourkaltseva, E. Pleteneva, O. Shaburova, S. Krylov, A. Karaulov, S. Zhavoronok, O. Svitich, V. Zverev, Phage phiKZ—The first of giants. *Viruses* **13**, 149 (2021).

72. H. Hama, T. Shimamoto, M. Tsuda, T. Tsuchiya, Characterization of a novel L-serine transport system in *Escherichia coli*. *J. Bacteriol.* **170**, 2236–2239 (1988).

73. S. Selvarasu, D. S.-W. Ow, S. Y. Lee, M. M. Lee, S. K.-W. Oh, I. A. Karimi, D.-Y. Lee, Characterizing *Escherichia coli* DH5α growth and metabolism in a complex medium using genome-scale flux analysis. *Biotechnol. Bioeng.* **102**, 923–934 (2009).

74. Y. Yang, A. M. Pollard, C. Höfler, G. Poschet, M. Wirtz, R. Hell, V. Sourjik, Relation between chemotaxis and consumption of amino acids in bacteria. *Mol. Microbiol.* **96**, 1272–1282 (2015).

75. M. D. Paepe, F. Taddei, Viruses' life history: Towards a mechanistic basis of a trade-off between survival and reproduction among phages. *PLoS Biol.* **4**, e193 (2006).

76. J. J. Barr, R. Auro, N. Sam-Soon, S. Kassegne, G. Peters, N. Bonilla, M. Hatay, S. Mourtada, B. Bailey, M. Youle, B. Felts, A. Baljon, J. Nulton, P. Salamon, F. Rohwer, Subdiffusive motion of bacteriophage in mucosal surfaces increases the frequency of bacterial encounters. *Proc. Natl. Acad. Sci. U.S.A.* **112**, 13675–13680 (2015).

77. J. Cremer, I. Segota, C. Yang, M. Arnoldini, J. T. Sauls, Z. Zhang, E. Gutierrez, A. Groisman, T. Hwa, Effect of flow and peristaltic mixing on bacterial growth in a gut-like channel. *Proc. Natl. Acad. Sci.* **113**, 11414–11419 (2016).

78. D. T. Fraebel, H. Mickalide, D. Schnitkey, J. Merritt, T. E. Kuhlman, S. Kuehn, Environment determines evolutionary trajectory in a constrained phenotypic space. *eLife* **6**, e24669 (2017).

79. M. Newville, T. Stensitzki, D. B. Allen, A. Ingargiola, LMFIT: Non-linear least-square minimization and curve-fitting for Python, Zenodo (2014); https://doi.org/10.5281/zenodo.598352.

80. J. J. Moré, "The Levenberg-Marquardt algorithm: Implementation and theory," in *Lecture Notes in Mathematics* (Berlin Springer Verlag, 1978), vol. 630, pp. 105–116.

81. F. C. Neidhardt, ed., *Escherichia Coli and Salmonella: Cellular and Molecular Biology* (ASM Press, ed. 2, 1996).

82. H. Hadas, M. Einav, I. Fishov, A. Zaritsky, Bacteriophage T4 development depends on the physiology of its host *Escherichia coli*. *Microbiology* **143**, 179–185 (1997).

83. L. You, P. F. Suthers, J. Yin, Effects of *Escherichia coli* physiology on growth of phage T7 in vivo and in silico. *J. Bacteriol.* **184**, 1888–1894 (2002).

84. J. Marchi, C. N. N. Minh, L. Debarbieux, J. S. Weitz, Multi-strain phage induced clearance of bacterial infections. *PLoS Comput. Biol.* **21**, e1012793 (2025).

85. P. Golec, J. Karczewska-Golec, M. Łoś, G. Węgrzyn, Bacteriophage T4 can produce progeny virions in extremely slowly growing *Escherichia coli* host: Comparison of a mathematical model with the experimental data. *FEMS Microbiol. Lett.* **351**, 156–161 (2014).

86. D. R. Roach, C. Y. Leung, M. Henry, E. Morello, D. Singh, J. P. Di Santo, J. S. Weitz, L. Debarbieux, Synergy between the host immune system and bacteriophage is essential for successful phage therapy against an acute respiratory pathogen. *Cell Host Microbe* **22**, 38–47.e4 (2017).
